# A novel two-step genome editing strategy with CRISPR-Cas9 provides new insights into telomerase action and *TERT* gene expression

**DOI:** 10.1186/s13059-015-0791-1

**Published:** 2015-11-10

**Authors:** Linghe Xi, Jens C. Schmidt, Arthur J. Zaug, Dante R. Ascarrunz, Thomas R. Cech

**Affiliations:** Howard Hughes Medical Institute, University of Colorado BioFrontiers Institute, Boulder, CO USA; Department of Molecular, Cellular & Developmental Biology, University of Colorado, Boulder, CO USA; Department of Chemistry & Biochemistry, University of Colorado, Boulder, CO USA

**Keywords:** Telomerase, TERT, Cajal body, cancer mutations, CRISPR-Cas9 genome editing, SNAP-tag

## Abstract

**Background:**

To facilitate indefinite proliferation, stem cells and most cancer cells require the activity of telomerase, which counteracts the successive shortening of telomeres caused by incomplete DNA replication at the very end of each chromosome. Human telomerase activity is often determined by the expression level of telomerase reverse transcriptase (TERT), the catalytic subunit of the ribonucleoprotein complex. The low expression level of TERT and the lack of adequate antibodies have made it difficult to study telomerase-related processes in human cells.

**Results:**

To overcome the low CRISPR-Cas9 editing efficiency at the *TERT* locus, we develop a two-step “pop-in/pop-out” strategy to enrich cells that underwent homologous recombination (HR). Using this technique, we fuse an N-terminal FLAG-SNAP-tag to TERT, which allows us to reliably detect TERT in western blots, immunopurify it for biochemical analysis, and determine its subcellular localization by fluorescence microscopy. TERT co-localizes detectably with only 5–7 % of the telomeres at a time in S-phase HeLa cells; no nucleolar localization is detected. Furthermore, we extend this approach to perform single base-pair modifications in the *TERT* promoter; reverting a recurrent cancer-associated *TERT* promoter mutation in a urothelial cancer cell line results in decreased telomerase activity, indicating the mutation is causal for telomerase reactivation.

**Conclusions:**

We develop a two-step CRISPR-Cas9 genome editing strategy to introduce precise modifications at the endogenous *TERT* locus in human cell lines. This method provides a useful tool for studying telomerase biology, and suggests a general approach to edit loci with low targeting efficiency and to purify and visualize low abundance proteins.

**Electronic supplementary material:**

The online version of this article (doi:10.1186/s13059-015-0791-1) contains supplementary material, which is available to authorized users.

## Background

All continuously proliferating cells, like stem cells and cancer cells, require a mechanism to compensate for telomere attrition during continuous division [1]. Most often this requirement is fulfilled by the telomerase enzyme. However, somatic cells lack telomerase activity, due to the transcriptional inactivation of the gene encoding TERT, the catalytic subunit of the telomerase holoenzyme [2–4]. Reactivation of *TERT* transcription in somatic cells allows them to divide indefinitely, which is a crucial step during tumorigenesis [5]. Therefore, investigating TERT expression is of great significance to understand how the level of telomerase activity is regulated under physiological and pathological conditions.

For several reasons, determining the expression level of TERT is hampered by the difficulty to detect the endogenous TERT protein. First, TERT is a lowly expressed protein with only several hundred molecules per cell [6]. Second, commercially available TERT antibodies have been shown to be either inefficient or non-specific in targeting endogenous TERT [6, 7]. CRISPR-Cas9-mediated genome editing provides an alternative approach, allowing tagging of the endogenous TERT protein with a well-defined epitope tag, for which well-characterized antibodies are available.

Furthermore, targeted genome editing also provides an approach to introduce specific mutations to the endogenous *TERT* locus and study their effects on TERT expression. For instance, two point mutations in the promoter region of the human *TERT* gene (*C-124T* and *C-146T*) have been reported to be highly recurrent in various cancers types [8, 9] and correlate with higher telomerase levels [10]. Results of luciferase reporter assay suggest that either mutation increases the transcriptional activity of the *TERT* promoter [8]. The association of these mutations with telomerase activation is well established, but the direct causality between these mutations and the activation of TERT expression in the endogenous context remains uncertain. Modifying the endogenous *TERT* promoter using genome editing can address this important question.

Here, we describe methods to modify the endogenous *TERT* locus with the CRISPR-Cas9 system, labeling the endogenous TERT protein with an affinity purification and localization tag or introducing a single base-pair modification in the *TERT* promoter. To overcome the low efficiency of genome editing at the *TERT* locus, we designed a two-step protocol similar to the “pop-in/pop-out” gene replacement method in yeast [11] to facilitate screening for successfully edited clones. With these methods, we generated HEK 293 and HeLa cell lines expressing FLAG-SNAP-tagged TERT protein, allowing efficient immunopurification (IP) and subcellular localization of endogenous TERT. Our results demonstrate that telomerase only localizes to a small number of telomeres at any given time. We also generated HEK 293T and SCaBER cells with a modified *TERT* promoter, suggesting that removing the *C-124T* mutation from a urothelial cancer cell line is sufficient to decrease the telomerase level and shorten telomeres. These methods not only provide useful tools for studying telomerase biology, but also offer a general approach to purify and visualize low abundance proteins, as well as making single base-pair modifications at genomic sites with low editing efficiency.

## Results

### Modification of the endogenous TERT protein with an N-terminal FLAG-SNAP-tag

**Fig. 1:**
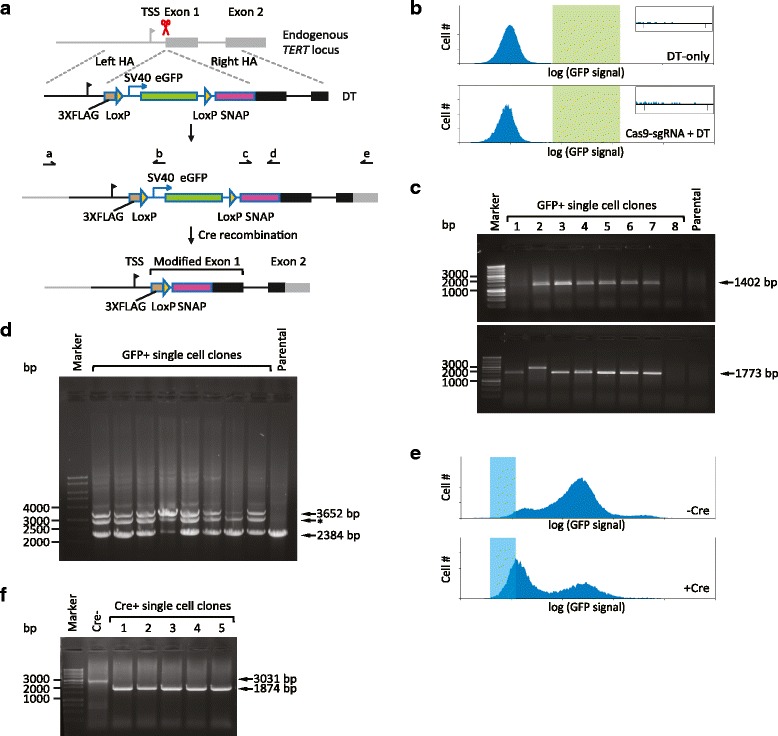
Inserting the sequence for the FLAG-SNAP-tag in the endogenous *TERT* locus. **a** Introducing an N-terminal FLAG-SNAP epitope tag to the endogenous TERT protein. First, a double-strand break was generated next to the translational start site of *TERT* with the CRISPR-Cas9 system (red scissors). Cells that underwent homologous recombination (HR) with the donor template (*DT*) containing the sequence for the tags and the SV40-driven enhanced green fluorescent protein (*eGFP*) expression cassette were screened for GFP signal and confirmed by PCR with primers *a*, *b*, *c*, and *e*, the sequences of which are listed in Table S3 in Additional file 1. *HA* homologous arm, *TSS* transcription start site. Next, the eGFP expression cassette was removed from the *TERT* locus through Cre-mediated recombination, leaving only the sequence for FLAG- and SNAP-tags and an intervening LoxP site at the 5′ end of the *TERT* coding sequence. **b** Fluorescence-activated cell sorting (FACS) screen for GFP-positive HEK 293 cells at the first step. Data shown are from cells transfected with the circular DT only or the circular DT + the Cas9-sgRNA plasmid. Cells with GFP signal in the *green-shaded region* were sorted. Inset figure: the green-shaded region enlarged. The DT-only group contained 0.5 % cells in this region and the Cas9-sgRNA + DT group contained 1.1 % cells in this region. **c** Sample data of PCR screen for clones that underwent HR. PCR products amplified by primer pairs a–b (*upper panel*) and c–e (*lower panel*), from genomic DNA of the GFP-positive HEK 293 single cell clones and untreated parental cells, were visualized by gel electrophoresis. Marker: 1 kb DNA ladder (Promega). Among the eight clones shown, six generated PCR products of the expected sizes in both PCRs (1402 bp for primer pair a–b, 1773 bp for primer pair c–e). Clone 2 only generated the correct PCR product in the a–b PCR. Clone 9 did not generate any PCR products, as was the case for the parental cells. **d** Sample data of PCR examination of the zygosity in the clones, from an experiment in which the sequence of a FLAG-tag and the eGFP expression cassette were inserted into the endogenous *TERT* locus in HEK 293T cells. PCR products amplified by primer pair a–e, from genomic DNA of the selected HEK 293T single cell clones and untreated parental cells, were visualized by gel electrophoresis. PCR with genomic DNA from the parental cells only generated the expected 2384 bp PCR product. Clones carrying the targeted insertion in both *TERT* alleles should generate only the 3652 bp PCR product. Clones carrying the targeted insertion in one of the *TERT* alleles should generate PCR products of both lengths. All eight clones shown were heterozygous for the insertion. The middle bands (indicated by asterisk) between the 3652 and 2384 bp bands might be the hybrid of one 2384 nucleotide strand and one 3652 nucleotide strand, since it contains the same sequence as the top band corresponding to the edited allele in our sequencing data. The same experiment was later performed on the HEK 293 clones with the insertion of FLAG-SNAP-tag sequence and the eGFP-expression cassette. The PCR product from the allele that underwent HR should be 4321 bp, which we failed to obtain, presumably because of the long length. We then regarded a clone as heterozygous if the 2384 bp product appeared. **e** FACS screen for GFP-negative cells at the second step. After transfection of the Cre plasmid or no transfection, cells with low GFP signal (*blue-shaded region*) were sorted. **f** Sample data of PCR screen for clones in which the eGFP expression cassette was excised through Cre recombination. PCR products amplified by primer pair a–d, from genomic DNA of the original GFP-positive clone without Cre expression (*Cre* ^*−*^) and several single cell GFP-negative clones after Cre recombination (*Cre* ^*+*^), were visualized by gel electrophoresis. Marker: 1 kb DNA ladder (Promega). In Cre^−^ cells, the size of the PCR product is 3031 bp. After the eGFP expression cassette is excised by Cre recombination, the size of the PCR product decreases to 1874 bp

We found that the efficiency of genome editing in the *TERT* 5′ region was very low (see below). We therefore designed a two-step protocol to introduce the sequence coding for a FLAG-SNAP-tag into the *TERT* locus (Fig. 1a). The tag was fused to the N-terminus of TERT because C-terminal tagging has been shown to impair the ability of telomerase to elongate telomeres within cells [12].

First, the tag sequence and an enhanced green fluorescent protein (eGFP) expression cassette flanked by LoxP-sites were inserted immediately upstream of the endogenous translational start site of *TERT* by transfecting a Cas9-single guide RNA (sgRNA) plasmid [13] and a donor template (DT) plasmid into cells (Fig. 1a). The fluorescence marker expressed from the eGFP cassette was used to screen for clones that had undergone homologous recombination (HR). We tested a number of sgRNA sequences (Table S1 in Additional file 1) in HEK 293T cells, and chose to use the sgRNA that directs Cas9 to cut between the base pairs at the −2 and −1 positions relative to the translational start site, because it would not target the DT, thus preventing cutting of an edited allele. We compared circular plasmid versus linearized plasmid as DT (Table S2 in Additional file 1) to determine whether there was an effect on targeting efficiency. The percentage of GFP-positive cells in the Cas9–sgRNA groups was higher than that in the corresponding control groups lacking Cas9–sgRNA. However, the targeting rates in both cases were low (~1 %), demonstrating the utility of the fluorescence marker. Because the frequency of non-specific integration (percentage of GFP-positive cells in the groups lacking Cas9–sgRNA) was higher using the linear DT, we chose to use the circular DT for subsequent experiments to reduce off-target effects.

The validated protocol was then used to edit HEK 293 cells, which carry two copies of the *TERT* gene. To isolate single cell clones, GFP-positive cells obtained by fluorescence-activated cell sorting (FACS) were seeded in 96-well plates by limiting dilution. The low efficiency of eGFP insertion at the *TERT* locus is readily apparent from FACS profiles of targeted cells (Fig. 1b). HR was confirmed by PCR with the primer pairs a–b and c–e (Fig. 1a, c; Table S3 in Additional file 1); 27 out of 32 clones (84 %) gave rise to PCR products of the expected size (Fig. 1c). To determine the number of *TERT* alleles that had undergone HR, we performed PCR with the primer pair a–e, demonstrating that all clones had undergone HR at one of the two alleles (Fig. 1d). We sequenced the PCR products of a–b, c–e and a–e from both alleles. The allele that had undergone HR carried the expected sequence, while the second allele in all clones contained small insertions or deletions (indels) around the Cas9 target site. This suggested that both alleles had been cut by Cas9, one being repaired by HR and the other being repaired by non-homologous end joining (NHEJ), generating small indels.

Next, we selected a single clone (clone 3 in Fig. 1c) to carry out the second step of the protocol. This clone contained a small deletion of 19 bp in the allele repaired by NHEJ (−1 to +18 relative to the original translation start site), removing the endogenous start codon and presumably knocking out TERT protein expression from this allele. We transiently expressed an eGFP-Cre fusion protein [14] in this clone to excise the eGFP cassette. Eight days after transfection, we screened the cell population for GFP-negative cells using FACS (Fig. 1e). Single cell clones were generated by limiting dilution, and PCR with primer pair a–d (Fig. 1a; Table S3 in Additional file 1) was used to confirm the excision of the eGFP expression cassette (Fig. 1f). All five clones tested were confirmed to contain the FLAG-SNAP-tag sequence inserted at the 5′ end of the endogenous *TERT* coding sequence. Gene copy number analysis verified that all clones contained two copies of *TERT* (Additional file 2).

An alternative approach with a puromycin resistance marker instead of the eGFP fluorescence marker in the DT plasmid was carried out in HeLa cells (Fig. S1 in Additional file 1). First, a puromycin-selected cell population was used to generate single cell clones. All isolated clones had undergone HR (Fig. S1a in Additional file 1). Two days after transient transfection of the eGFP-Cre plasmid, single cell clones were seeded from the GFP-positive cell population. All clones generated with this method had undergone successful excision of the puromycin resistance cassette (Fig. S1b in Additional file 1). Sequencing of the PCR products verified the FLAG-SNAP-tagged alleles that had undergone HR, and revealed untagged alleles that contained small indels around the Cas9 cut site (presumably via NHEJ). Since HeLa cells carry five or six copies of the *TERT* gene [10], the exact number of HR alleles and NHEJ alleles is unknown.

### Analysis of expression and activity of the FLAG-SNAP-TERT protein

**Fig. 2:**
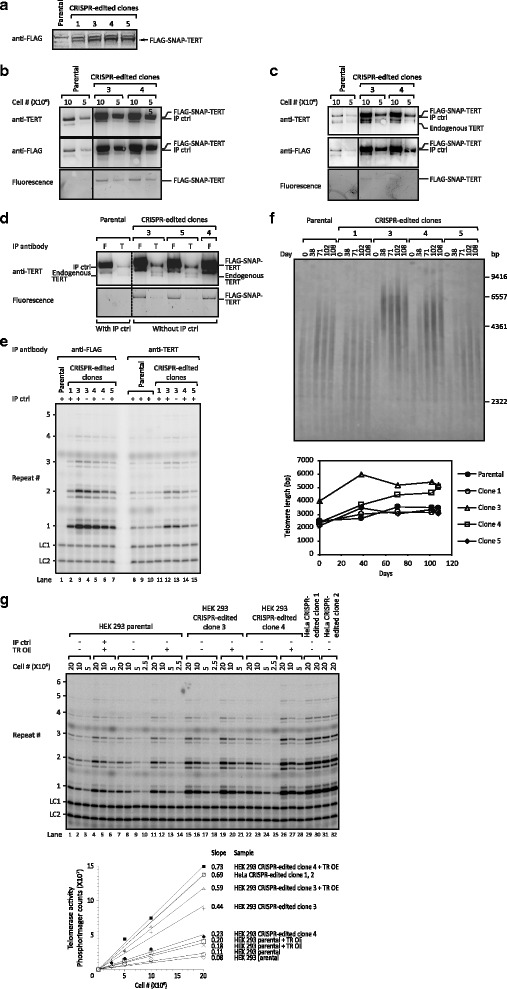
Expression, purification and activity of the FLAG-SNAP-TERT protein. **a** Western blot of lysates of parental HEK 293 cells and edited clones expressing FLAG-SNAP-TERT using a FLAG antibody. Bands at the expected size of FLAG-SNAP-TERT (*arrow*) indicate that the fusion protein is expressed in the edited clones. **b** FLAG-SNAP-TERT immunopurified with a FLAG antibody from parental HEK 293 cells and various edited clones was revealed by western blot using antibodies against the FLAG-tag and TERT. Fluorescence was detected using SNAP Surface® 594 dye covalently bound to the SNAP-tag. All samples were supplemented with IP control (*IP ctrl*). **c** FLAG-SNAP-TERT immunopurified with a TERT-antibody from parental HEK 293 cells and various edited clones was revealed by western blot using antibodies against the FLAG-tag and TERT. A band with the size of endogenous TERT was also observed on the western blot of the modified clones, which should not arise from the NHEJ allele as the start codon was removed by the indel. One possibility is that the original start codon for the endogenous TERT on the FLAG-SNAP allele was still used to some extent instead of the start codon before the FLAG-tag sequence. Fluorescence detection and IP ctrl is as in panel (b). **d** Western blots and fluorescence imaging, comparing the IP efficiency of FLAG-SNAP-TERT using the anti-FLAG (*F*) or anti-TERT (*T*) antibody. **e** Direct telomerase activity assay of telomerase purified from parental HEK 293 cells and various edited HEK 293 clones with the anti-FLAG or anti-TERT antibody. Note lack of telomerase activity in lane 1 because no FLAG-labeled TERT is expressed in the parental cells. *LC1* and *LC2* are two oligonucleotide loading controls. **f** Telomeric restriction fragment length analysis by Southern blot of parental HEK 293 cells and several edited clones expressing FLAG-SNAP-TERT. Cells were harvested at the indicated time points. Average telomere lengths are plotted in the lower panel. Clones 3 and 4, which have higher levels of telomerase activity as shown in panel (e), have elongated telomeres. **g** Direct telomerase activity assay of telomerase purified from parental HEK 293 cells and various edited HEK 293 and HeLa clones using the anti-TERT antibody. Cells were transfected with a telomerase RNA expression plasmid (*TR OE*) to assure that FLAG-SNAP-TERT was the limiting component for telomerase assembly. IP ctrl was included in one sample after cell lysis to confirm that it did not affect telomerase activity. *LC1* and *LC2* are two oligonucleotide loading controls. Quantitative analysis of the data is plotted in the lower panel

To determine whether the FLAG-SNAP-TERT fusion protein was expressed in the edited HEK 293 cells, we analyzed whole cell lysates by western blot. An anti-FLAG antibody detected a band of the expected size in the edited clones, but not in the parental HEK 293 cells (Fig. 2a), confirming the expression of the FLAG-SNAP-TERT protein. Similarly, FLAG-SNAP-TERT was only detected in the edited HeLa clones but not in the parental HeLa cells (Fig. S2a in Additional file 1).

Next, we compared IP of FLAG-SNAP-TERT using a FLAG antibody with that using a well-established TERT antibody, which is useful for IP but not for western blots [15, 16]. As an internal control for the IP we added catalytically inactive ProA-FLAG-TERT (IP control (IP ctrl)). The ProA-FLAG-tag is smaller than the FLAG-SNAP-tag, and thus IP ctrl runs at an intermediate size between endogenous TERT and FLAG-SNAP-TERT on SDS-PAGE. Anti-FLAG IPs efficiently enriched FLAG-SNAP-TERT from the edited HEK 293 clones but not from the parental cells (Fig. 2b; Fig. S2b in Additional file 1). (Most of the signal in lanes 3–6 of Fig. 2b is due to the FLAG-SNAP-TERT, because the IP ctrl is present at low levels as seen in lanes 1–2.) The identity of the FLAG-SNAP-TERT band was verified by its detection with both anti-FLAG and anti-TERT antibodies (Fig. 2b). Both antibodies also readily detected the IP ctrl added to the parental HEK293 cells, which do not contain FLAG-SNAP-TERT. Additionally, FLAG-SNAP-TERT was detectable using fluorescence imaging, indicating that the SNAP-tag is fully functional (Fig. 2b). Similar results were obtained using the edited HeLa clones (Fig. S2b in Additional file 1). IP with the TERT antibody also purified FLAG-SNAP-TERT from the edited clones but not the parental cells (Fig. 2c; Fig. S2c in Additional file 1). In addition, the TERT antibody also enriched endogenous TERT from the parental cells. Curiously, wild-type (WT) TERT bands were also present in the edited clones, potentially due to usage of the WT *TERT* start codon.

To compare the IP efficiency of the FLAG and TERT antibodies, we carried out IPs without adding the IP ctrl to the edited clones (IP ctrl was still included with parental cell samples, since it is clearly distinguishable from endogenous TERT). In all edited cell lines, the anti-FLAG IP was much more efficient for purifying TERT (compare each pair of F and T lanes in Fig. 2d). Quantification of the fluorescence signal indicated that the IP with the anti-FLAG antibody purified five- to seven-fold higher amounts of FLAG-SNAP-TERT (Fig. 2d). Thus, the anti-FLAG IP provides a more efficient purification of TERT expressed from an endogenous chromosomal locus than previous methods.

To determine the expression level of FLAG-SNAP-TERT in the edited clones relative to endogenous TERT, we compared the amounts of TERT and FLAG-SNAP-TERT purified using the anti-TERT IP (Fig. 2d; samples from edited clones did not include IP ctrl). Unexpectedly, FLAG-SNAP-TERT in edited clones was present at approximately 20-fold higher levels than endogenous TERT in parental cells, even though FLAG-SNAP-TERT is expressed from its endogenous locus. Reverse transcription-quantitative PCR (RT-qPCR) analysis indicated the edited clones expressed higher levels of TERT mRNA than the parental cells (Fig. S2d in Additional file 1), suggesting that the sequence of the FLAG-SNAP-tag improved *TERT* transcription and/or TERT mRNA stability.

Finally, we tested the enzymatic activity of FLAG-SNAP-TERT to determine whether the tag interfered with the catalytic function of TERT. Telomerase purified using the FLAG and TERT antibodies was used in direct telomerase extension assays, measuring incorporation of radioactive dGTP into a telomeric oligonucleotide primer. As expected, anti-TERT IP resulted in telomerase activity from all cells, while anti-FLAG IP showed telomerase activity only in the edited clones (Fig. 2e; Fig. S2e in Additional file 1). In the edited clones, the FLAG antibody precipitated four- to eight-fold more telomerase activity than the TERT antibody, consistent with the increased efficiency of TERT IP (Fig. 2d, e). Furthermore, in the edited clones 3 and 4, IP with the TERT antibody yielded two- to five-fold more telomerase activity than in the parental cells, consistent with TERT being overexpressed. Clones 1 and 5 did not show elevated telomerase activity; the reason for this clonal variation is unknown. Accordingly, we observed telomere lengthening in clones 3 and 4 but not clones 1 and 5 (Fig. 2f). Activity in edited HeLa clones was comparable to the increased activity in HEK 293 clones 3 and 4 (Fig. 2g). The observation that the fold increase in the telomerase activity (less than five-fold) is much lower than the fold-increase in the protein level (20-fold) is presumably because the amount of telomerase RNA (TR), the other key component of the telomerase catalytic core, limits telomerase assembly. This was anticipated from the endogenous molecule numbers of TERT and TR previously measured [6], and was also indicated by transient overexpression of TR in the edited clones, which further increased their telomerase activity (Fig. 2g).

In summary, these results demonstrate that the FLAG-SNAP-tag allows a higher IP efficiency and retains the enzymatic activity of TERT.

### Subcellular localization of FLAG-SNAP-TERT

The localization of TERT in human cells has been inferred from many studies with overexpressed tagged protein [17–20]. Although cytolocalization of endogenous TERT has been reported [21, 22], such studies have been frustrated by the low abundance of TERT and lack of a good antibody. Here, we use FLAG-SNAP-TERT expressed from the endogenous locus as a surrogate for endogenous TERT, realizing that the overexpression due to insertion of the tag, although much more modest than that produced by standard transient transfection, could nevertheless influence the results. CRISPR-edited HeLa clones were used for these experiments because HeLa cells attach to the glass surface better than HEK 293 cells. We labeled the FLAG-SNAP-TERT using a cell-permeable substrate for the SNAP-tag (BG-647-SiR) [23]. Cells were fixed and stained for TRF2 and coilin to visualize telomeres and Cajal bodies, respectively.

**Fig. 3:**
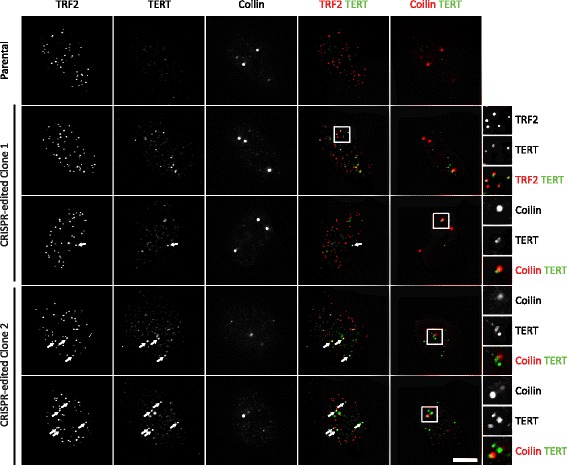
Subcellular localization of FLAG-SNAP-TERT. IF analysis of fixed HeLa cells expressing FLAG-SNAP-TERT. The SNAP-tag was labeled with SNAP-Cell® 647-SiR dye (scale bar = 5 μm). Telomeres and Cajal bodies were stained with antibodies against TRF2 and coilin, respectively. Edited cells but not parental cells showed FLAG-SNAP-TERT foci that co-localized with telomeres and Cajal bodies. Two independent clones expressing FLAG-SNAP-TERT were used to generate the images shown

**Fig. 4:**
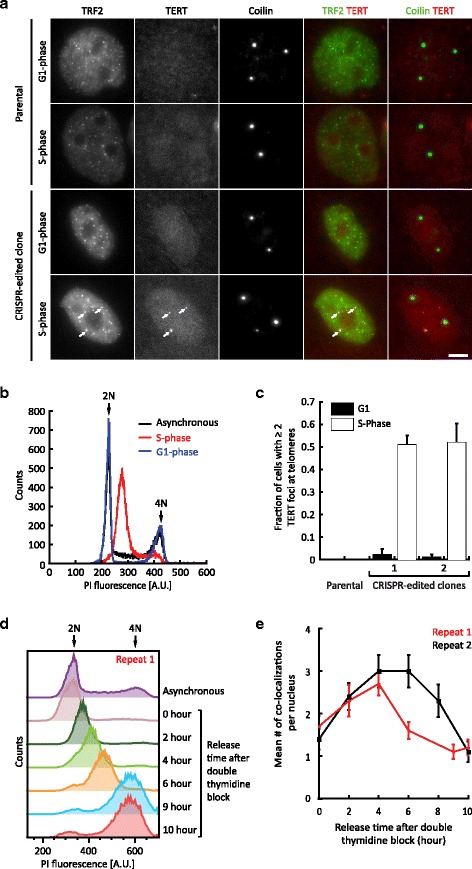
TERT localizes to telomeres in S but not G1 phase of the cell cycle. **a** IF analysis of fixed HeLa cells expressing FLAG-SNAP-TERT, synchronized in G1 and S phase of the cell cycle (scale bar = 5 μm). Cells expressing FLAG-SNAP-TERT displayed telomere-localized TERT foci in S but not G1 phase of the cell cycle, while parental cells never showed telomere-localized TERT foci. **b** FACS analysis of the DNA content of cells synchronized in S phase showed a peak between the 2 N and 4 N peaks of asynchronous cells, confirming that they were in S phase. The G1 cell population contained 2 N and 4 N peaks but was depleted for cells with intermediate DNA content. 4 N cells, which failed to release from their mitotic arrest, were easily distinguished from G1 cells by their morphology. **c** Quantification of the number of TERT foci which co-localized with TRF2 signals in edited HeLa cells synchronized at different stages of the cell cycle. Data were generated from two independent experiments, each analyzing 50 cells per condition (mean ± standard deviation). **d** FACS analysis of the DNA content of edited HeLa cells released from a double thymidine block as they transition through S phase. Prior to release, the cell population contained mostly cells with 2 N DNA content, which progressively increased as the cells underwent DNA replication. Nine to ten hours after release, DNA replication was complete, as indicated by the majority of cells having 4 N DNA content. **e** Quantification of the number of TERT foci co-localized with TRF2 signals at different time points during S phase (50 cells per time point, mean ± standard error of the mean; for corresponding images see Fig. S4 in Additional file 1). *A.U.* arbitrary units, *Propidium Iodide (PI)*

Cells containing FLAG-SNAP-TERT displayed bright foci at telomeres and Cajal bodies, the expected localization sites of TERT. No nucleolar staining of TERT was observed, in contrast to previous immunofluorescence (IF) experiments using an anti-TERT antibody [17, 21]. The parental HeLa cells showed only background staining (Fig. 3; for full field-of-view images see Data files 3–6 in Additional file 3). Z-stacks of the cells were also analyzed to assure the co-localization (Fig. S3a in Additional file 1). Since telomerase is recruited to telomeres during S phase of the cell cycle [21, 24], we compared cells synchronized in S phase and G1 phase (Fig. 4a–c; for full field-of-view images see Data files 3, 7 and 8 in Additional file 3). We found each S-phase nucleus contained approximately 40 TRF2 foci (Fig. S3b in Additional file 1), instead of the 150–300 telomeres present in a HeLa cell pre- and post-replication, respectively. Thus, the foci presumably contain clusters of around four to eight telomeres on average. Most S-phase cells contained a small fraction of telomere clusters that co-localized with TERT (~5 %). These results suggest that only a small number of telomeres are being elongated at any given time point, even in S phase.

To further analyze telomere recruitment of telomerase during S phase, we synchronized edited HeLa cells at the G1/S border using a double thymidine block and visualized TERT localization to telomeres as cells progressed through S phase (Fig. 4d). TERT localization increased throughout the first 4–6 hours of S phase, at which point it reached its maximal frequency of a mean of around three co-localizations per cell, which corresponded to ~7 % of TRF2 foci (Fig. 4e; Fig. S4 in Additional file 1; for full field-of-view images see Data files 3, 9-14 in Additional file 3). After completion of DNA synthesis (9–10 hours post-release), TERT localization dropped to its minimum (mean of around one co-localization per cell), indicating that telomerase dissociates from telomeres in G2 phase of the cell cycle. These observations are consistent with previous results obtained using fluorescence in situ hybridization (FISH) for TR, the RNA component of telomerase [21].

Thus, the SNAP-tag enables subcellular localization of the lowly expressed TERT, and its localization at biologically functional sites demonstrates that TERT function is not impaired by the N-terminal FLAG-SNAP-tag or by the modest overexpression. The number of Cajal bodies per cell (typically one to four; Fig. S3c in Additional file 1) was indistinguishable from that observed in the parental HeLa cells, in contrast to cells with highly overexpressed telomerase where unnatural neo-Cajal bodies associate with every telomere [19, 20]. It is worth noting antibodies against the FLAG-epitope did not detect FLAG-SNAP-TERT at telomeres, due to background foci (Data file 3, 15 in Additional file 3), indicating that the SNAP labeling is more specific than conventional IF labeling.

### Single base-pair modification in the endogenous *TERT* promoter

**Fig. 5:**
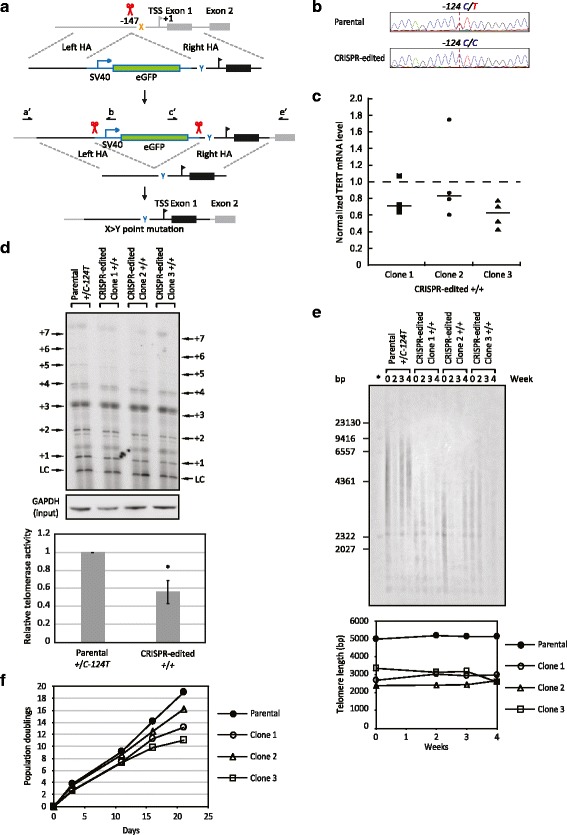
Single base-pair modification at the endogenous *TERT* promoter. **a** Outline of the protocol to modify a single base pair at the endogenous *TERT* promoter. First, a double-strand break was generated at the endogenous *TERT* promoter next to the targeted base pair with the CRISPR-Cas9 system (*red scissors*). Cells that underwent HR with the DT containing the SV40-driven eGFP expression cassette and the single base-pair modification were screened by GFP signal and confirmed by PCR with primers a’, b, c’, and e’, the sequences of which are listed in Table S3 in Additional file 1. *HA* homologous arm, *TSS* transcription start site. Next, the eGFP expression cassette was excised by two double-strand breaks generated by the CRISPR-Cas9 system. Cells that underwent HR with the DT containing the modified *TERT* promoter sequence were screened based on the loss of the fluorescent signal and sequencing the PCR products generated with primers a’ and e’. **b** Sample sequencing data demonstrating the reversion of the *C-124T* mutation from the *TERT* promoter in SCaBER cells. Peak color: *red*, T; *green*, A; *blue*, C; *black*, G. **c** TERT mRNA levels in the modified SCaBER clones are plotted relative to that in the parental cells (1.0, *dashed line*), as was measured by RT-qPCR with GAPDH mRNA as an internal control. The *black line* in each group of data points indicates the median value. As a group, the three clones had significantly reduced mRNA levels relative to the parental cells (*p* = 0.04 by Wilcoxon rank-sum test). **d** Telomerase activity in the modified SCaBER clones was lower than that in the parental cells, as measured by the IP-direct extension assay (*LC* loading control). Signal of the extension products on the gel was normalized to the LC signal and GAPDH protein levels in the IP input, which were quantified by western blot. The quantitative result is shown in the bar graph (mean ± standard deviation, n = 3 biological replicates, *p < 0.05 by Student’s t-test). **e** Telomeric restriction fragment length analysis by Southern blot in the parental cells and modified clones. Cells were harvested at the indicated time points. The average telomere length in each sample is plotted in the lower panel. *λ DNA-HindIII digest markers. **f** Growth curves for the parental cells and modified clones

Recently, two highly recurrent point mutations were identified in the *TERT* promoter in multiple cancers and shown to be associated with telomerase activation during tumorigenesis. To explore the effects of these mutations on TERT expression levels in the endogenous context, we extended our two-step “pop-in/pop-out” strategy to modify single base pairs in the *TERT* promoter (Fig. 5a). A single base-pair substitution was first introduced into the *TERT* promoter alongside an eGFP expression cassette, which was then removed by a second round of CRISPR-mediated editing, resulting in a *TERT* promoter with only a single base-pair alteration.

The protocol was first tested using HEK 293T cells, which contain WT *TERT* promoter sequences. Ten sgRNA sequences were tested, and a sequence directing Cas9 to cut between the −148 and −147 positions relative to the endogenous translational start site was chosen based on its targeting efficiency and position of cleavage (Table S1 and Fig. S5 in Additional file 1). The DT for the first step contained the *C-146T* mutation and an eGFP cassette inserted between base pairs −140 and −139, which disrupted the sgRNA recognition site. On days 6, 14 and 20 post-transfection, three rounds of FACS were carried out to enrich for the GFP-positive cells, from which single cell clones were generated. PCR with the primer pair a’–b or c’–e’ (Fig. 5a; Table S3 in Additional file 1) was used to identify clones that had undergone HR. PCR with the primer pair a’–e’ suggested that both *TERT* alleles had undergone HR (homozygous) in two clones, while in another three clones only one *TERT* allele had undergone HR (heterozygous) (Fig. S6a in Additional file 1). Sequencing of the PCR products revealed that two out of the three heterozygous clones had a small insertion of two cytidines between −148 and −147 in the allele that had not undergone HR. Quantification of the TERT mRNA levels demonstrated that TERT expression was reduced to 10 % and 60 % of WT level in the homozygous and heterozygous clones, respectively, indicating that the insertion of the eGFP expression cassette in the *TERT* promoter disrupted transcription (Fig. S6b in Additional file 1). The decrease in the TERT mRNA levels correlates with the decrease in the levels of TERT protein and telomerase activity, as well as telomere shortening (Fig. S6c, d in Additional file 1).

We then used a heterozygous clone (clone 5) to carry out the second step of the protocol, removing the eGFP cassette. Two Cas9-sgRNA plasmids targeting the edges of the eGFP cassette (Table S1 in Additional file 1) were co-transfected with the DT plasmid, which contained the sequence of *TERT* locus from −589 to +353, and included the *C-146T* point mutation. Eight days post-transfection, the cell population was analyzed by FACS. The Cas9^−^ group (transfected with only the DT) contained ~11.7 % GFP-negative cells, which could be due to epigenetic silencing of the eGFP expression cassette. The Cas9^+^ group contained a higher percentage of GFP-negative cells (~16.5 %). The GFP-negative cells in the Cas9^+^ group were used to generate single cell clones. Eighty clones were screened by PCR with the primer pair a’–e’ and sequencing, among which two were identified that had undergone HR to remove the eGFP expression cassette and had incorporated the *C-146T* mutation (Fig. S6e in Additional file 1). Copy number variation analysis verified that both clones have two copies of *TERT*. The remaining clones either contained small indels or had lost one copy of the *TERT* gene (Additional file 2). TERT mRNA levels in the two *C-146T* clones measured by RT-qPCR were not higher than that in the parental HEK 293T cells (Fig. S6f in Additional file 1). However, since TERT expression is activated through an alternative mechanism in HEK 293T cells, it is perhaps not surprising that the introduction of the *TERT* promoter mutation did not lead to a further increase of TERT expression.

To determine the effects of the *TERT* promoter mutations in a context where they would be functional, we used the established protocol to modify the *TERT* promoter in SCaBER, a urothelial cancer cell line which contains two *TERT* alleles, one of which carries the *C-124T* mutation [10]. Based on the hypothesis that the *C-124T* allele is more active in transcription than the *WT* allele, we targeted the *C-124T* allele to revert the mutation, since it has been suggested that the CRISPR-Cas9 system has increased accessibility in transcriptionally active regions [25]. A DT with the eGFP cassette inserted in the WT promoter sequence was used for the first step of editing. Out of 18 GFP-positive clones generated, 16 (89 %) contained the eGFP cassette inserted into the *TERT* locus. Based on sequencing results, we identified a clone in which the *C-124T* allele had undergone HR while the WT allele had neither undergone HR nor contained indels. This clone was subjected to the second round of genome editing to remove the eGFP cassette. Out of 200 clones analyzed by PCR and sequencing, three were found to contain only the WT *TERT* promoter sequence (Fig. 5b). Copy number variation analysis demonstrated that all three clones had two copies of *TERT* (Additional file 2), ruling out the possibility that the mutant *TERT* allele was lost during editing.

To determine the effect of reverting the promoter mutation on TERT transcription, we analyzed TERT mRNA levels by RT-qPCR. The three clones exhibited reduced mRNA levels relative to the parental SCaBER cells (Fig. 5c). To assess whether the reduced TERT transcription had functional consequences, we tested the telomerase activity in these modified clones with direct telomerase extension assays following TERT IP. The cellular telomerase activity levels were decreased by 40–50 % in the modified clones compared with the parental cells (Fig. 5d). Consistent with these results, telomere shortening was observed in the edited clones (Fig. 5e). Furthermore, all clones grew at a slower rate compared with the parental cells (Fig. 5f). These results suggest that the *C-124T* mutation in the SCaBER cell line is necessary for full activation of TERT expression. Reverting the mutation in the *TERT* promoter decreases telomerase levels and telomere length, and thereby limits the growth of these cancer cells.

## Discussion

We developed several two-step “pop-in/pop-out” strategies for precise genome editing at the endogenous *TERT* locus, which allowed us to label the TERT protein with an N-terminal epitope tag and facilitated single base-pair modifications in the *TERT* promoter. Using cells edited with this strategy, we achieved detection of TERT protein and IP of telomerase with unprecedented efficiency, and we demonstrated that telomerase was only detectable at 5–7 % of telomeres at any time in S phase. We also corrected a cancer-associated *TERT* promoter mutation in a urothelial cancer cell line and showed that the telomerase level and growth rate of these cancer cells were decreased.

### Strategies for precise genome editing at endogenous loci with low targeting efficiency

CRISPR-Cas9-mediated genome editing provides an easy and rapid way to introduce targeted modifications in the human genome. However, targeting efficiencies vary greatly among different loci [26]. When we attempted to utilize CRISPR-Cas9-mediated genome editing to study telomerase biology, we found *TERT* to be one of the loci with a low targeting efficiency. Because *TERT* is not a very actively transcribed gene, its chromatin environment might prevent the access of the Cas9–sgRNA complex. Another potential explanation is that the high GC content around the *TERT* 5′ region might block target recognition by the Cas9–sgRNA complex — for example, because the DNA was difficult to melt or because it formed higher-order structures like G-quadruplexes. To overcome this hurdle, we employed a “pop-in/pop-out” approach similar to that used in yeast genome editing, including a marker to enrich for clones that had undergone HR. Screening for fluorescent cells (or selecting for drug-resistant cells) greatly enriched the desired clones in the first step; over 80 % of the GFP-positive clones had undergone HR. It should be pointed out that careful analysis of the zygosity, sequence and copy number of the targeted gene is crucial, as we found that a large percentage of the clones that had one *TERT* allele modified by HR contained small indels in the other allele, which was presumably also cut by Cas9 but then repaired through NHEJ. We also observed that some clones lost a copy of *TERT* after editing, most likely due to the telomere-proximal location of *TERT* causing partial chromosome loss after the induction of a double-strand break.

Parallel experiments using a circular plasmid or a linear plasmid as the DT demonstrated that their efficiency of integration through HR is similar (circular, 1.0 %; linear, 1.2 %; Table S2 in Additional file 1). However, the linear plasmid is more frequently integrated into off-target sites in the genome. We therefore chose the circular DT because the ratio of specific to non-specific DT integration in the GFP-positive clones was higher.

During the “pop-out” step, the marker expression cassette was removed through Cre-mediated recombination, in the case of introducing the FLAG-SNAP-tag. A LoxP site remained between the FLAG- and the SNAP-coding sequences, but it did not interfere with the function of either tag. To generate single base-pair modifications, a second round of CRISPR-Cas9-mediated editing was necessary. Loss of the fluorescence marker was used to enrich for clones that had eliminated the eGFP expression cassette, and 1.5 % of the selected clones contained the desired sequence alteration. The frequency and specificity of our approach might be further improved with previously described methods such as using the D10A Cas9 nickase, genetically or chemically inhibiting the NHEJ pathway, or cell cycle synchronization [27–30].

An unanticipated observation was that the insertion of the FLAG-SNAP-tag sequence increased the expression of the *TERT* gene. As mentioned above, *TERT* has a very specific sequence context around its 5′ region, which is presumably responsible for its low transcriptional activity. The insertion of the FLAG-LoxP-SNAP sequence may somehow interrupt this state and improve the transcription level. In general, C-terminal tagging might be less likely to alter the transcription level. However, C-terminal tagging of TERT impairs its function within cells [12] and therefore was not an option for our studies. In addition, the SNAP-tag could function as a solubility tag, stabilizing the TERT protein. In the future, different tags could be tested, in order to identify ones that have little effect on TERT expression. Importantly, the total amount of assembled telomerase holoenzyme was only increased less than five-fold based on our telomerase activity measurements. Therefore, the modification of TERT with the FLAG-SNAP-tag should only have minimal effects in telomerase biology.

### Telomerase distributed unevenly among telomeres at S phase

Using the FLAG-SNAP-TERT clones, we analyzed the subcellular localization of the endogenously produced TERT protein. As expected, TERT localized to Cajal bodies and telomeres. Previous studies using anti-TERT antibodies also detected non-telomere associated foci [21] and signals associated with nucleoli [17, 21, 22], which were not seen in our experiments and may reflect a lack of specificity of IF detection of TERT. In contrast to overexpressed telomerase, which localizes to all the telomeres detected in S phase [19], the endogenously produced TERT was only detectable at 5–7 % of TRF2 foci in S phase, which could represent clusters of multiple telomeres recruiting telomerase. This observation indicates that the endogenous telomerase only elongates a limited number of telomeres at any given time point. Previous studies have suggested that all telomeres in human cancer cells are elongated in each cell cycle [31], which requires telomerase recruitment to each telomere. In budding yeast, telomerase does not extend all telomeres in any given cell cycle, but rather only the shortest telomeres [32]; consistent with this finding, only a subset of telomeres co-localize with yeast TR during S phase [33]. Our data in human cells are consistent with recruitment of telomerase to a subset of telomeres at any given time point in S phase, but we cannot rule out that a small number or a single telomerase holoenzyme localizes to each telomere, because our method is not sensitive enough to detect single molecules.

If telomerase is recruited to only a small number of telomeres at any given time point, sequential telomere elongation would have to occur to add telomeric repeats to many or all telomeres in a single cell cycle. Our data are consistent with the model in which telomeres sequentially recruit telomerase, potentially controlled by the replication timing of individual telomeres, which differs dramatically among chromosomes [34]. In agreement with this notion, TERT localization to telomeres is maximal in the middle of S phase. Importantly, recruitment of telomerase to telomeres does not necessarily have to lead to telomere elongation. Telomerase might be recruited to every telomere in a single S phase but only elongate a subset of them. Several studies have suggested that the shortest telomeres are preferentially elongated in human cells [35, 36], but in these cases telomerase was overexpressed, which has been shown to deregulate telomerase recruitment to telomeres [19]. Alternatively, our data are also consistent with only a subset of telomeres being elongated in a single cell cycle. To distinguish these models, time-lapse microscopy of telomerase recruitment to telomeres throughout S phase will be necessary.

### Reverting a cancer-associated *TERT* promoter mutation decreased telomerase levels and limited the growth rate of cancer cells

Recent studies of various human cancer genomes have identified two highly recurrent *TERT* promoter mutations (*C-124T* and *C-146T*) [8, 9, 37–39], which generate new binding sites for the ETS transcription factor GABP [40]. Luciferase reporter gene assays comparing WT/mutant *TERT* promoter sequences suggested that promoters with either of the two mutations drive higher levels of gene expression (approximately two-fold increase compared with WT) [8]. Quantitative analysis of endogenous TERT expression levels, cellular telomerase activity and telomere lengths in cancer cell lines with or without these mutations has demonstrated that these mutations are associated with higher levels of telomerase and longer telomere lengths [10]. To test the causality between the mutations and endogenous telomerase levels, we edited the endogenous *TERT* promoter, allowing us to make comparisons among presumptively isogenic cell lines. Reverting the *C-124T* mutation in a urothelial cancer cell line, which originally carried one WT allele and one *C-124T* mutant allele, resulted in decreased telomerase levels, telomere shortening and reduced proliferation rate of these cells.

One caveat of our genome editing protocol is that the expression level of TERT affects cell growth. Both steps (insertion of the eGFP cassette in the promoter and introducing C at position −124 instead of T) would decrease the TERT expression level and render the desired clones at a selective disadvantage. This would greatly decrease the success rate of getting a desired clone; and it also led to the concern that the three SCaBER clones we obtained might have collected other mutations that counteract the effects of loss of the *C-124T* mutation and increase telomerase levels. This raises a general question about how to target genes that affect cell growth. One potential solution would be to supply a functional copy of the gene during the editing process and remove it afterwards. In addition, any potential off-target effects of the genome editing could also affect the phenotypes of clones. To help control for the off-target effects, one could introduce both WT and mutant promoter sequences to replace the eGFP expression cassette integrated in the first step of genome editing. Due to these potential concerns, we realize the phenotypes we observed might reflect more than just the loss of the *TERT* promoter mutation.

Interestingly, introducing the *C-146T* mutation in HEK 293T cells, which originally carried two WT alleles, was insufficient to increase the level of telomerase. In our HEK 293T cell genome editing, a small insertion of two cytidines was incorporated in the allele that underwent NHEJ; it is possible that this insertion reduced the transcription of *TERT*, which neutralized the activation effect of the *C-146T* mutation on the allele that underwent HR. However, the fact that clones with one CC-insertion allele and one eGFP cassette-insertion allele have ~40 % of the telomerase level compared with parental cells suggested the CC-insertion does not greatly affect TERT expression (Fig. S6 in Additional file 1). Another possibility is that the *C-124T* mutation and the *C-146T* mutation function differently. But a more likely explanation would be that, because HEK 293T cells have already activated TERT expression through an alternative mechanism, the rate-limiting step that can be facilitated by the promoter mutation has already been surmounted by other means. Whatever the mechanism, these results highlight the importance of genomic background in the functional investigation of disease-related mutations.

## Conclusions

The 5′ region of the human *TERT* gene has a very low efficiency for CRISPR-Cas9 genome editing. This limitation was overcome by devising two-step “pop-in/pop-out” editing strategies, either screening for the insertion of an eGFP gene or selecting for insertion of a puromycin-resistance gene. In one set of experiments, insertion of a FLAG-SNAP-tag at the N-terminus of TERT allowed reliable detection of the protein, efficient IP of active telomerase ribonucleoprotein complexes, and cytolocalization. Typically, only 5–7 % of clustered telomeres showed detectable telomerase co-localization at any one time in S phase. One possible explanation is that telomerase is recruited to telomeres sequentially rather than simultaneously. Another set of experiments introduced single base-pair changes in the *TERT* gene promoter at sites of recurrent cancer-specific mutations, which had previously been found to be associated with increased telomerase activity. Reverting the heterozygous *C-124T* mutation in a urothelial cancer cell line suggested the causality of the mutation for telomerase activity, telomere length maintenance and cell growth rate. More generally, our studies show how introducing protein tags at endogenous loci with genome editing can overcome multiple constraints with purifying and visualizing low-abundance proteins.

## Materials and methods

### Cell culture

HEK 293 cells (ATCC), HEK 293T cells (ATCC) and HeLa-EM2-11ht cells (Tet Systems Holdings GmbH and Co. KG) were grown in high glucose Dulbecco’s modified Eagle medium (DMEM) supplemented with 10 % fetal bovine serum (FBS), 2 mM GlutaMAX™-I (Life Technologies), 100 units/ml penicillin and 100 μg/ml streptomycin at 37 °C with 5 % CO_2_. SCaBER cells, a gift from D. Theodorescu (University of Colorado Cancer Center, Denver), were grown in minimum essential medium (MEM) supplemented with 10 % FBS, 2 mM GlutaMAX^TM^-I, 0.1 mM minimal non-essential amino acids (Life Technologies), 1 mM sodium pyruvate, 100 units/ml penicillin and 100 μg/ml streptomycin at 37 °C with 5 % CO_2_.

### Plasmid construction and transfection

sgRNA sequences (Table S1 in Additional file 1) were cloned individually into the pX330 plasmid vector as described in [13]. DT sequences with the eGFP/puro-resistance expression cassette for both methods were synthesized by GENEWIZ and cloned into the pUC57-Kan plasmid. The DT with the endogenous *TERT* sequence from −589 to +353 was PCR amplified from the HEK 293T genomic DNA and cloned into the pUC57-Kan plasmid. The QuickChange II XL Site-Directed Mutagenesis Kit (Agilent Technologies, 200522) was used to modify the DT plasmids with corresponding mutations. The sequences of all the DT plasmids are provided as Data files 16-19 in Additional file 4. pBS598 EF1alpha-EGFPcre plasmid [14] was purchased from Addgene.

The Nucleofector™ 2b Device (Lonza) was used to introduce pX330 Cas9-sgRNA plasmids and pUC67-Kan DT plasmids into cells. For HEK 293 and HEK 293T cells, Amaxa™ Cell Line Nucleofector™ Kit V was used as transfection reagent according to the manufacturer’s instructions. For each transfection, one million cells were transfected with 2.5 μg pX330 Cas9-sgRNA plasmids and 2.5 μg pUC67-Kan DT plasmids. For SCaBER cells, Cell Line Optimization Nucleofector™ Kit for Nucleofector™ Device was first used to optimize the transfection conditions. Based on the results, Amaxa™ Cell Line Nucleofector^TM^ Kit V and Program L-029 were chosen. For each transfection, 0.5 million cells were transfected with 2.5 μg pX330 Cas9-sgRNA plasmids and 2.5 μg pUC67-Kan DT plasmids. For HeLa cells, one million cells were transfected with 1.0 μg pX330 Cas9-sgRNA plasmids and 1.0 μg pUC67-Kan DT plasmid using Lipofectamine® 2000 Reagent (Invitrogen) according to the manufacturer’s instructions. The pBS598 EF1alpha-EGFPcre plasmid was transfected with Lipofectamine® 2000 into both HEK 293 and HeLa cells.

### FACS

Trypsinized cells were spun down at 200 g for 5 min and re-suspended in phosphate-buffered saline (PBS) with 3 % bovine serum albumin (BSA). The cells were sorted based on the GFP signal on a MoFlo™ XDP cell sorter (Beckman).

### Genomic DNA extraction, genotyping and copy number variation analysis

Genomic DNA samples were prepared with QuickExtract™ DNA Extraction Solution (Epicentre) or GenElute™ Mammalian Genomic DNA Miniprep Kit (Sigma-Aldrich) according to the manufacturers’ instructions. Sequences of the *TERT* locus were PCR amplified for Sanger sequencing with the corresponding primers (Table S3 in Additional file 1) as described in [10]. Copy number variation analysis was performed on a StepOne™ Real-Time PCR instrument (Life Technologies) using FAM-labeled TaqMan™ Assays as described in [10].

### RNA extraction and RT-qPCR

Total RNA samples were extracted with TRIzol® Reagent (Ambion) according to the manufacturer’s instructions. For SCaBER, cells were first treated with 0.5 μg/μl proteinase K in TE buffer (10 mM Tris–HCl pH 8.0, 1 mM EDTA) at 65 °C for 20 min and then homogenized by TRIzol®. The extracted total RNA samples were treated with RQ1 RNase-free DNase (Promega) to eliminate genomic DNA contamination. cDNA was then prepared using the High Capacity cDNA Reverse Transcription kit (Applied Biosystems). RT-qPCR was performed with iQ™ SYBR® Green Supermix (Bio-Rad) on the LightCycler® 480 Real-Time PCR System (Roche) with primers described in [6].

### Telomerase IP and telomerase activity assay

FLAG IP was performed with Anti-FLAG® M2 Affinity Gel (Sigma-Aldrich, A2220) on HEK 293/HeLa cell lysates prepared with CHAPS lysis buffer (10 mM Tris–HCl pH 7.5, 1 mM MgCl_2_, 1 mM EGTA, 0.5 % CHAPS, 10 % glycerol, 1 mM phenylmethanesulfonyl fluoride, 1 mM dithiothreitol). Telomerase IP with the sheep polyclonal anti-TERT antibody, which was a gift from S. Cohen (Children’s Medical Research Institute and University of Sydney, Sydney, Australia) and telomerase activity assay were performed as described in [6, 15].

### Western blot and SNAP-tag fluorescence labeling

The protein samples were electrophoresed on a 4–12 % Bis-Tris gel (Life Technologies), followed by standard western blotting protocols. Primary antibodies used were as follows: anti-TERT antibody (Abcam, ab32020, 1:1000), anti-GAPDH antibody (Santa Cruz, sc-137179, 1:1000), anti-FLAG horseradish peroxidase-conjugated antibody (Sigma-Aldrich, A8592, 1:1000). Secondary antibodies used were as follows: peroxidase-AffiniPure donkey anti-rabbit IgG (H + L) (Jackson, 711-035-152, 1:5000), peroxidase-AffiniPure donkey anti-mouse IgG (H + L) (Jackson, 715-035-150, 1:5000). SuperSignal® West Pico Chemiluminescent Substrate (Thermo Scientific) was used to generate signals on western blots. The signals were detected with a FluorChem HD2 imaging system (Alpha Innotech) and quantified with ImageQuant TL v2005 software. To detect the SNAP-tag, 10 μM SNAP Surface® 594 (New England Biolabs, S9112S) was added to the input samples at the beginning of the IP. The fluorescence signals were detected with a Typhoon Trio PhosphorImager (GE Healthcare) and quantified using ImageQuant TL v2005 software.

### Telomeric restriction fragment length analysis

For each sample, 1.5 μg genomic DNA was digested with Hinf1 and Rsa1 at 37 °C for over 4 h and then electrophoresed on a 0.8 % agarose-1× TBE gel at 70 V for a total of 1100 V-h together with a 5′ end ^32^P-labeled λ DNA-HindIII digest ladder. Next, the gel was shaken in the following solutions: 0.25 M HCl for 15 min, 0.5 M NaOH–1.5 M NaCl for two rounds of 15 min and 0.5 M Tris–1.5 M NaCl pH 7.5 for 30 min. Then the DNA was transferred from the gel to the Hybond™-N^+^ membrane (GE Healthcare) by capillary blotting with 20× SSC buffer (3 M NaCl, 300 mM sodium citrate, pH 7.0), and cross-linked to the membrane under UV 254 nm at 1200 × 100 μJ/cm^2^. The membrane was pre-hybridized in Rapid-hyb buffer (GE Healthcare) at 35 °C for 30 min, then hybridized in Rapid-hyb buffer with 5′ end ^32^P-labeled (TTAGGG)_3_ probe at 35 °C for 1 h. After that, the membrane was washed three times with 0.1× SSC, 0.1 % SDS at 50 °C for 20 min each time. The signals on the membrane were detected with the Typhoon Trio PhosphorImager and quantified with ImageQuant TL v2005 software.

### Cell growth rate analysis

Parental SCaBER cells and the three modified clones were grown in T75 flasks. At each passage, the total cell number in each flask was counted, and 0.1 million cells were seeded in fresh growth medium. The cells were not confluent during the culturing.

### IF and live cell SNAP-labeling

The SNAP-tag was labeled using 1–2 μM SNAP-Cell® 647-SiR (New England Biolabs, S9102S) for 2–3 h in DMEM containing 10 % FBS, 2 mM GlutaMAX^TM^-I, 100 units/ml penicillin and 100 μg/ml streptomycin at 37 °C with 5 % CO_2_. Following SNAP-labeling, cells were washed with PBS and pre-extracted with Triton X Buffer (20 mM HEPES pH 7.9, 50 mM NaCl, 3 mM MgCl_2_, 300 mM sucrose, 0.5 % Triton X-100), for 1 min on ice, rinsed with PBS and fixed with formaldehyde (4 % formaldehyde, 2 % sucrose in PBS) for 10 min at room temperature. Cells were then re-permeabilized using Triton X Buffer for 10 min at room temperature, and incubated in blocking buffer (3 % BSA in PBS) for 30 min. After blocking, cells were incubated with primary antibodies for TRF2 (Imgenex, IMG-124A, 1:500) and coilin (Santa Cruz, sc-32860, 1:100) in blocking buffer for 1 h. Next, cells were washed with PBS and incubated with secondary antibodies (Life Technologies, A-31556, and Abcam, ab150117, 1:500) in blocking buffer for 1 h. After a final wash, cells were mounted using ProLong® Diamond Antifade Mountant (Life Technologies, P36970).

### Cell cycle synchronization and flow cytometry analysis

For S-phase synchronization, cells were arrested in growth medium containing 2 mM thymidine for 16 h. To release the cells, they were washed three times with PBS and cultured in regular growth medium for 3 h. For the double thymidine block, cells were released for 9 h, followed by a second thymidine arrest prior to release into S phase as described above. To enrich for cells in the G1 phase of the cell cycle, cells were arrested in mitosis using growth medium supplemented with 100 ng/ml nocodazole for 14–16 h. To release the cells into G1, the mitotic cell population was washed three times with PBS and cultured in regular growth medium. To determine the cell cycle distribution of the cell populations, cells were fixed in 70 % ethanol and subsequently stained with propidium iodide solution (0.2 mg/ml RNase A, 0.1 % Triton X-100, 20 μg/ml propidium iodide in PBS). Stained cells were analyzed using the FACScan sorter (Becton-Dickinson).

### Microscopy

All images were acquired on a Deltavision Core deconvolution microscope (Applied Precision) using a 60× 1.42NA PlanApo N (Olympus) or 100× UPLanSApo 1.4NA (Olympus) objective and a sCMOS camera. Twenty Z-sections with 0.2 μm spacing were acquired for each image with identical exposure conditions within each experiment. For presentation in figures, representative images were deconvolved (where indicated), followed by generation of maximum intensity projections of 5–10 Z-sections, which were scaled identically for all experimental conditions.

## Additional files

Additional file 1:
**Supplemental Figures S1–S6 and Tables S1–S3.** (PDF 12143 kb) Additional file 2:
**A figure showing the** ***TERT*** **copy number analysis for various cell lines generated in this study.** (PDF 226 kb) Additional file 3:
**Data file 3, is a detailed description of data files 4-15.** Data files 4-15, which are maximum intensity projections of images for all cell biological experiments, including images of an experiment using a FLAG antibody to detect FLAG-SNAP-TERT in HeLa cells. (ZIP 43134 kb) Additional file 4:
**Data files 16-19, which are sequences of all the DT plasmids, as indicated by their names.** Plasmids will be deposited to Addgene. For the single base-pair modification step 1/2 DT (Data files 4 and 5 in Additional file 3), the −124 and −146 positions were modified with the C > T mutations based on the detailed situation. (ZIP 13 kb)

## Abbreviations

bp: base pair
BSA: bovine serum albumin
DMEM: Dulbecco’s modified Eagle medium
DT: donor template
eGFP: enhanced green fluorescent protein
FACS: fluorescence-activated cell sorting
FBS: fetal bovine serum
HR: homologous recombination
IF: immunofluorescence
indel: small insertions or deletion
IP: immunopurification
NHEJ: non-homologous end joining
PBS: phosphate buffered saline
RT-qPCR: reverse transcription-quantitative polymerase chain reaction
sgRNA: single guide RNA
TERT: telomerase reverse transcriptase
TR: telomerase RNA
WT: wild type

## References

[1] Meyerson M (2000). Role of telomerase in normal and cancer cells. J Clin Oncol.

[2] Lingner J, Hughes TR, Shevchenko A, Mann M, Lundblad V, Cech TR (1997). Reverse transcriptase motifs in the catalytic subunit of telomerase. Science.

[3] Kilian A, Bowtell DD, Abud HE, Hime GR, Venter DJ, Keese PK (1997). Isolation of a candidate human telomerase catalytic subunit gene, which reveals complex splicing patterns in different cell types. Hum Mol Genet.

[4] Gunes C, Lichtsteiner S, Vasserot AP, Englert C (2000). Expression of the hTERT gene is regulated at the level of transcriptional initiation and repressed by Mad1. Cancer Res.

[5] Shay JW, Bacchetti S (1997). A survey of telomerase activity in human cancer. Eur J Cancer.

[6] Xi L, Cech TR (2014). Inventory of telomerase components in human cells reveals multiple subpopulations of hTR and hTERT. Nucleic Acids Res.

[7] Wu YL, Dudognon C, Nguyen E, Hillion J, Pendino F, Tarkanyi I (2006). Immunodetection of human telomerase reverse-transcriptase (hTERT) re-appraised: nucleolin and telomerase cross paths. J Cell Sci.

[8] Huang FW, Hodis E, Xu MJ, Kryukov GV, Chin L, Garraway LA (2013). Highly recurrent TERT promoter mutations in human melanoma. Science.

[9] Horn S, Figl A, Rachakonda PS, Fischer C, Sucker A, Gast A (2013). TERT promoter mutations in familial and sporadic melanoma. Science.

[10] Borah S, Xi L, Zaug AJ, Powell NM, Dancik GM, Cohen SB (2015). Cancer. TERT promoter mutations and telomerase reactivation in urothelial cancer. Science.

[11] Scherer S, Davis RW (1979). Replacement of chromosome segments with altered DNA sequences constructed in vitro. Proc Natl Acad Sci U S A.

[12] Banik SS, Guo C, Smith AC, Margolis SS, Richardson DA, Tirado CA (2002). C-terminal regions of the human telomerase catalytic subunit essential for in vivo enzyme activity. Mol Cell Biol.

[13] Cong L, Ran FA, Cox D, Lin S, Barretto R, Habib N (2013). Multiplex genome engineering using CRISPR/Cas systems. Science.

[14] Le Y, Miller JL, Sauer B (1999). GFPcre fusion vectors with enhanced expression. Anal Biochem.

[15] Cohen SB, Graham ME, Lovrecz GO, Bache N, Robinson PJ, Reddel RR (2007). Protein composition of catalytically active human telomerase from immortal cells. Science.

[16] Cohen SB, Reddel RR (2008). A sensitive direct human telomerase activity assay. Nat Methods.

[17] Yang Y, Chen Y, Zhang C, Huang H, Weissman SM (2002). Nucleolar localization of hTERT protein is associated with telomerase function. Exp Cell Res.

[18] Wong JM, Kusdra L, Collins K (2002). Subnuclear shuttling of human telomerase induced by transformation and DNA damage. Nat Cell Biol.

[19] Zhong FL, Batista LF, Freund A, Pech MF, Venteicher AS, Artandi SE (2012). TPP1 OB-fold domain controls telomere maintenance by recruiting telomerase to chromosome ends. Cell.

[20] Schmidt JC, Dalby AB, Cech TR. Identification of human TERT elements necessary for telomerase recruitment to telomeres. Elife. 2014;3. doi: 10.7554/eLife.03563. 10.7554/eLife.03563 PMC4359370 25271372

[21] Tomlinson RL, Ziegler TD, Supakorndej T, Terns RM, Terns MP (2006). Cell cycle-regulated trafficking of human telomerase to telomeres. Mol Biol Cell.

[22] Abreu E, Aritonovska E, Reichenbach P, Cristofari G, Culp B, Terns RM (2010). TIN2-tethered TPP1 recruits human telomerase to telomeres in vivo. Mol Cell Biol.

[23] Lukinavicius G, Umezawa K, Olivier N, Honigmann A, Yang G, Plass T (2013). A near-infrared fluorophore for live-cell super-resolution microscopy of cellular proteins. Nat Chem.

[24] Jady BE, Richard P, Bertrand E, Kiss T (2006). Cell cycle-dependent recruitment of telomerase RNA and Cajal bodies to human telomeres. Mol Biol Cell.

[25] Wu X, Scott DA, Kriz AJ, Chiu AC, Hsu PD, Dadon DB (2014). Genome-wide binding of the CRISPR endonuclease Cas9 in mammalian cells. Nat Biotechnol.

[26] Yu Z, Ren M, Wang Z, Zhang B, Rong YS, Jiao R (2013). Highly efficient genome modifications mediated by CRISPR/Cas9 in Drosophila. Genetics.

[27] Ran FA, Hsu PD, Lin CY, Gootenberg JS, Konermann S, Trevino AE (2013). Double nicking by RNA-guided CRISPR Cas9 for enhanced genome editing specificity. Cell.

[28] Maruyama T, Dougan SK, Truttmann MC, Bilate AM, Ingram JR, Ploegh HL (2015). Increasing the efficiency of precise genome editing with CRISPR-Cas9 by inhibition of nonhomologous end joining. Nat Biotechnol.

[29] Chu VT, Weber T, Wefers B, Wurst W, Sander S, Rajewsky K (2015). Increasing the efficiency of homology-directed repair for CRISPR-Cas9-induced precise gene editing in mammalian cells. Nat Biotechnol.

[30] Lin S, Staahl BT, Alla RK, Doudna JA (2014). Enhanced homology-directed human genome engineering by controlled timing of CRISPR/Cas9 delivery. Elife.

[31] Zhao Y, Sfeir AJ, Zou Y, Buseman CM, Chow TT, Shay JW (2009). Telomere extension occurs at most chromosome ends and is uncoupled from fill-in in human cancer cells. Cell.

[32] Teixeira MT, Arneric M, Sperisen P, Lingner J (2004). Telomere length homeostasis is achieved via a switch between telomerase- extendible and -nonextendible states. Cell.

[33] Gallardo F, Laterreur N, Cusanelli E, Ouenzar F, Querido E, Wellinger RJ (2011). Live cell imaging of telomerase RNA dynamics reveals cell cycle-dependent clustering of telomerase at elongating telomeres. Mol Cell.

[34] Arnoult N, Schluth-Bolard C, Letessier A, Drascovic I, Bouarich-Bourimi R, Campisi J (2010). Replication timing of human telomeres is chromosome arm-specific, influenced by subtelomeric structures and connected to nuclear localization. PLoS Genet.

[35] Fakhoury J, Marie-Egyptienne DT, Londono-Vallejo JA, Autexier C (2010). Telomeric function of mammalian telomerases at short telomeres. J Cell Sci.

[36] Britt-Compton B, Capper R, Rowson J, Baird DM (2009). Short telomeres are preferentially elongated by telomerase in human cells. FEBS Lett.

[37] Killela PJ, Reitman ZJ, Jiao Y, Bettegowda C, Agrawal N, Diaz LA (2013). TERT promoter mutations occur frequently in gliomas and a subset of tumors derived from cells with low rates of self-renewal. Proc Natl Acad Sci U S A.

[38] Weinhold N, Jacobsen A, Schultz N, Sander C, Lee W (2014). Genome-wide analysis of noncoding regulatory mutations in cancer. Nat Genet.

[39] Vinagre J, Almeida A, Populo H, Batista R, Lyra J, Pinto V (2013). Frequency of TERT promoter mutations in human cancers. Nat Commun.

[40] Bell RJ, Rube HT, Kreig A, Mancini A, Fouse SD, Nagarajan RP (2015). Cancer. The transcription factor GABP selectively binds and activates the mutant TERT promoter in cancer. Science.

