# Supplementary material for: A novel two-step genome editing strategy with CRISPR-Cas9 provides new insights into telomerase action and TERT gene expression
Source: Genome Biol. 2015 Nov 10;16:231. doi: 10.1186/s13059-015-0791-1 (PMC4640169; doi:10.1186/s13059-015-0791-1)
Supplement: Additional file 1: — Supplemental Figures S1–S6 and Tables S1–S3. (PDF 12143 kb) [file 13059_2015_791_MOESM1_ESM.pdf]

Figure S1. Generating CRISPR-edited HeLa clones with FLAG-SNAP-labelled endogenous TERT.

(A) PCR products amplified from genomic DNA of HeLa cells transfected with a Cas9-sgRNA plasmid and a DT plasmid, which contained the FLAG-SNAP-tag sequence and the expression cassette of a puromycin resistance marker, analogous to the DT outlined in Fig. 1A. Primer pair a-b was used to amplify the fragment upon the occurrence of HR, as done in Fig. 1C. Arrow: expected PCR product produced by HR. (B) PCR products of HeLa clones with the sequence of the FLAG-SNAP-tag inserted at the *TERT* locus, with or without the treatment with Cre to remove the puromycin resistance expression cassette. PCR was performed with the primer pair a-d, as done in Fig. 1F. Arrow: expected PCR products.

Figure S2. Analysis of the expression of FLAG-SNAP-TERT.

(A) Western blot of cell lysates prepared from parental HeLa cells and HeLa clones expressing FLAG-SNAP-TERT, probed with a FLAG-antibody. Edited clones but not the parental HeLa cells showed a band of the expected size corresponding to FLAG-SNAP-TERT. (B) FLAG-SNAP-TERT immunopurified with a FLAG-antibody from parental and edited HEK 293/HeLa cells was revealed by western blot using a FLAG-antibody. No IP ctrl was added. Edited cells but not parental cells showed a band on the western blot, which is also detected by fluorescence labelling, indicating that FLAG-SNAP-TERT is efficiently purified and fluorescently labelled. (C) Western blot of FLAG-SNAP-TERT immunopurified with a TERT-antibody from CRISPR-edited HeLa clones. (D) TERT mRNA levels in the edited HEK 293 clones examined by RT-qPCR, with 18S rRNA as an internal control. (E) Direct telomerase activity assay of telomerase purified from parental and edited HEK 293/HeLa cells with a FLAG-antibody. Note lack of telomerase activity in Lane 1 in both gels because no FLAG-labelled TERT is expressed in the parental cells. LC 1 and 2: two oligonucleotide loading controls.

Figure S3. Subcellular localization of FLAG-SNAP-TERT, telomeres and Cajal bodies in S-phase.

(A) IF analysis of fixed HeLa cells expressing FLAG-SNAP-TERT, synchronized in S-phase of the cell cycle. The SNAP-tag was labelled with SNAP-Cell® 647-SiR dye and telomeres were stained with TRF2. All images were analysed as Z-stacks to assure that telomere-localized TERT foci were in the same Z-plane as the corresponding TRF2 foci. Three Z-planes are shown and co-localization is only detectable in one of the Z-slices (Scale bar = 5 μm). (B) Quantification of the number of TRF2 foci in cells synchronized

in S-phase (mean ± S. D., n=10). (C) Quantification of the number of coilin foci in cells synchronized in S-phase (mean ± S. D., n=100).

Figure S4. Subcellular localization of FLAG-SNAP-TERT during different time points in S-phase. IF analysis of fixed HeLa cells expressing FLAG-SNAP-TERT at different time points during S-phase after release from a double thymidine block. The SNAP-tag was labelled with SNAP-Cell® 647-SiR dye, telomeres and Cajal bodies were stained with TRF2 and coilin respectively (Scale bar = 5 μm).

Figure S5. Targeting efficiency comparison among the sgRNA sequences targeting the *TERT* promoter. HEK 293T cells were transfected with different Cas9-sgRNA plasmids and the same linear DT containing an eGFP expression cassette inserted between base pairs -140 and -139 in the sequence of *TERT* promoter. On day 4 after the transfection, GFP-positive cells were sorted from each population for further culturing, which were then analysed on day 15 after the transfection by flow cytometry. The figure shows the percentage of GFP-positive cells in each population at the second round of cell sorting. Dashed line: background level of GFP-positive cells in the DT-only group.

Figure S6. Examination of the CRISPR-edited HEK 293T clones.

(A) Genotype of the *TERT* promoter in the clones with the inserted SV40-driven eGFP expression cassette. PCR was performed with primer pair a'-e' (Fig. 5A, Table S3). The endogenous *TERT* sequence should generate a PCR product of 1110 bp. The insertion of the eGFP expression cassette increases the size of the PCR product to 2262 bp. (*: suspected hybrid of one 1110 nt strand and one 2262 nt strand). Based on the PCR results and sequencing of the products, the genotypes of the clones are summarized in the lower panel. Marker: 1 kb DNA ladder (Promega). The green box stands for the eGFP expression cassette. Among the five clones, 1 and 4 were homozygous for the insertion of the eGFP cassette; 2, 3 and 5 were heterozygous for the insertion; and 2 and 5 had a small insertion of 2 cytidines in the allele without the insertion of the eGFP cassette. (B) TERT mRNA levels in the five clones in Panel A were measured by RT-qPCR, with GAPDH mRNA as an internal control (mean ± S. D., n = 4 technical replicates). (C) TERT protein levels and telomerase activity in the five clones in Panel A were examined by TERT IP, follow by western blot and direct extension assay. GAPDH and histone H3 protein in the input lysates were quantified by western blot as internal controls. Telomerase activity in the input lysates

and elution samples was quantified by the direct extension assay. LC: loading control. (D) Telomeric restriction fragment length analysis by Southern blot in the parental cells and modified clones in Panel A. Cells were harvested at the indicated time points. (*: λ DNA-HindIII digest markers) (E) Sample sequencing data demonstrating the introduction of the *C-146T* mutation in the *TERT* promoter of HEK 293T. The trace file shows the superposition of two sequences, arising from the two *TERT* alleles in the cells. Allele 1 contained the *C-146T* single base-pair mutation (*). Allele 2 contained a CC insertion between base pairs -148 and -147 (*-148CC*) (boxed), which resulted from the first step of editing. We also had sequencing data from the other direction and together they demonstrated the *C-146T* and *-148CC* are in different alleles. (F) TERT mRNA levels in the *C-146T*/*-148CC* insertion clones were examined by RT-qPCR, with GAPDH mRNA as an internal control (mean ± S. D., n = 2 biological replicates).

Table S1. sgRNA sequences tested and utilized.

The first 5 sgRNAs were tested for the insertion of the FLAG-SNAP-tag sequence and the eGFP expression cassette before the endogenous start codon of *TERT*, among which the one cutting between -2 and -1 was chosen. The middle 10 sgRNAs were tested for the insertion of the eGFP expression cassette in the *TERT* promoter, among which the one cutting between -148 and -147 was chosen. The last 2 were used to remove the eGFP expression cassette from the *TERT* promoter.

Table S2. Comparison between the circular DT and the linear DT.

HEK 293T cells were transfected with the corresponding plasmid(s) as indicated. On day 8 after the transfection, the cell populations were analyzed by flow cytometry. The percentage of GFP-positive cells in each population is listed.

Table S3. Sequences of the primers used in the work.

The positions of primer a, b, c, d, e, a’, e’ are marked in Fig. 1A and 5A. The sequencing primer was used for the Sanger sequencing analysis examining the sequences of *TERT* promoter.

Table S1. Sequences of sgRNAs.

Cutting position relative to the translational start site of *TERT*	sgRNA (5' to 3')
Between -61 and -60	gctcctcgcggcgcgagtttc
Between -33 and -32	gctgcgtcctgctgcgcacgt
Between -2 and -1	ggggagcgcgcggcatcgcg
Between +18 and +19	gagcgcacggctcggcagcg
Between +55 and +56	gctgcgcagccactaccgcg
Between -175 and -174	gccagctccgcctcctccgcg
Between -174 and -173	gacggggcggggtccgcgcgg
Between -157 and -156	gacccgggaggggtcgggacg
Between -148 and -147	gcgccccgtcccgacccctcc
Between -145 and -144	gctgggccggggacccggga
Between -140 and -139	gcccgacccctcccgggtccc
Between -125 and -124	gtccccggcccagccccctc
Between -122 and -121	ggggctgggagggcccggag
Between -121 and -120	gaggggctgggagggcccgga
Between -97 and -96	ggcggggccgcggaaaggaa
At the upstream boundary of the eGFP expression cassette	gattccacagggtcgaccacc
At the downstream boundary of the eGFP expression cassette	gtcttcggacctcgcggccc

Table S2. Percentage of GFP-positive HEK 293T cells 10 days after the transfection of the Cas9-sgRNA plasmid and a circular/linear DT plasmid.

Experimental Group	Percentage of GFP+ cells (%)
Circular DT only	0.9
Circular DT + Cas9-sgRNA	1.9
Linear DT only	2.2
Linear DT + Cas9-sgRNA	3.4

Table S3. Primer sequences.

Primer	Forward (5' to 3')
a	ccctgcaaggcctcgggaga
b	actttccacacctggttgctgac
c	atggacaaagattgcgaaat
d	ggatcctggcgcgcctatac
e	tggtctccacgagcctccga
a'	cgtccagggagcaatgcgt
c'	cggcatggacgagctgtacaagt
e'	acgctggtggtgaaggcctc
Sequencing primer	ctggcgtccctgcaccctgg

Figure S1

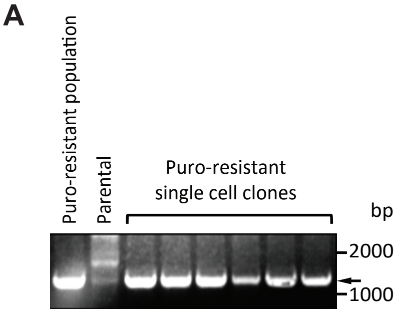
A
Puro-resistant population
Parental
Puro-resistant single cell clones
bp
2000
1000

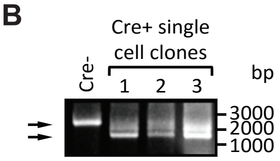
B
Cre+ single cell clones
Cre-
1
2
3
bp
3000
2000
1000

Figure S2

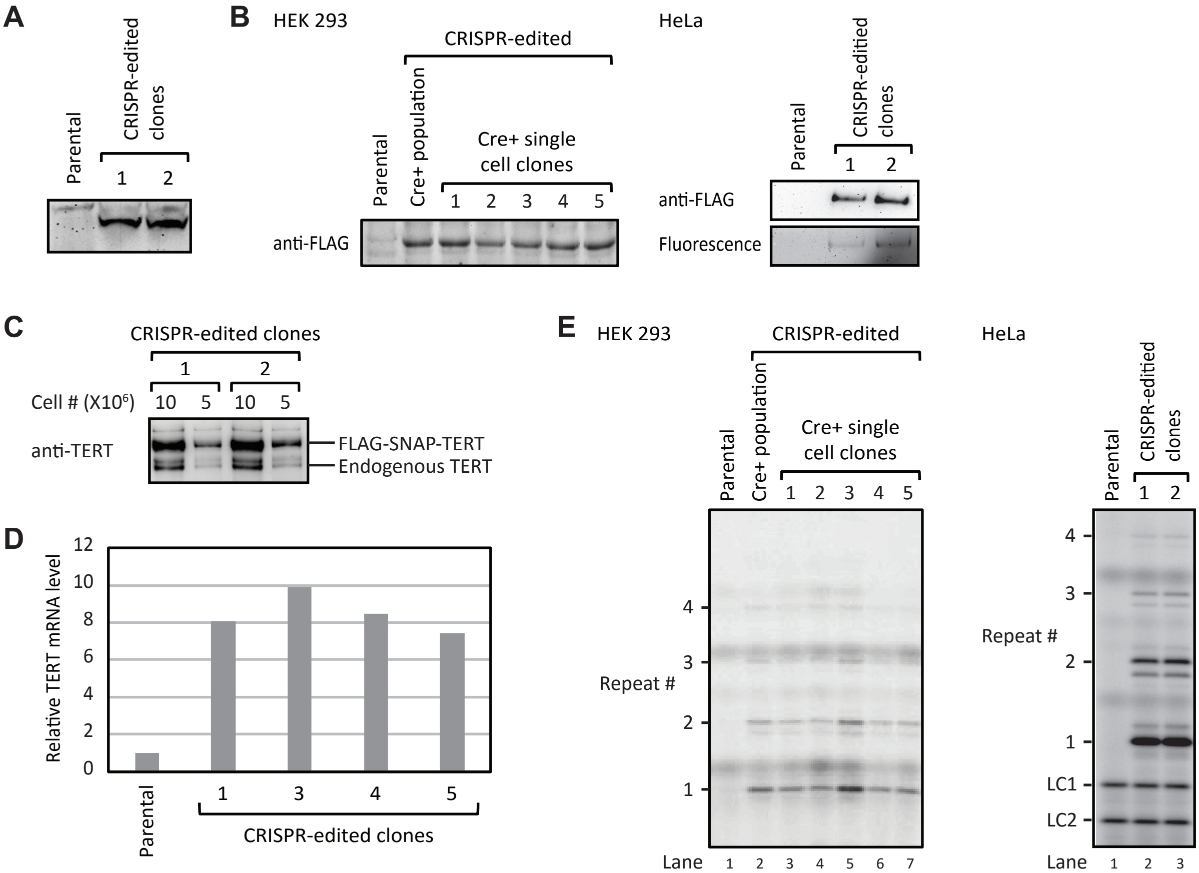

A
Parental
CRISPR-edited clones
1
2
B
HEK 293
CRISPR-edited
Parental
Cre+ population
Cre+ single cell clones
1 2 3 4 5
anti-FLAG
HeLa
Parental
CRISPR-editied clones
1 2
anti-FLAG
Fluorescence
C
CRISPR-edited clones
1
2
Cell # (X10^6)
10 5 10 5
anti-TERT
FLAG-SNAP-TERT
Endogenous TERT
D
Relative TERT mRNA level
0
2
4
6
8
10
12
Parental
1 3 4 5
CRISPR-edited clones
E
HEK 293
CRISPR-edited
Parental
Cre+ population
Cre+ single cell clones
1 2 3 4 5
Repeat #
4
3
2
1
Lane 1 2 3 4 5 6 7
HeLa
Parental
CRISPR-editied clones
1 2
Repeat #
4
3
2
1
LC1
LC2
Lane 1 2 3

Figure S3

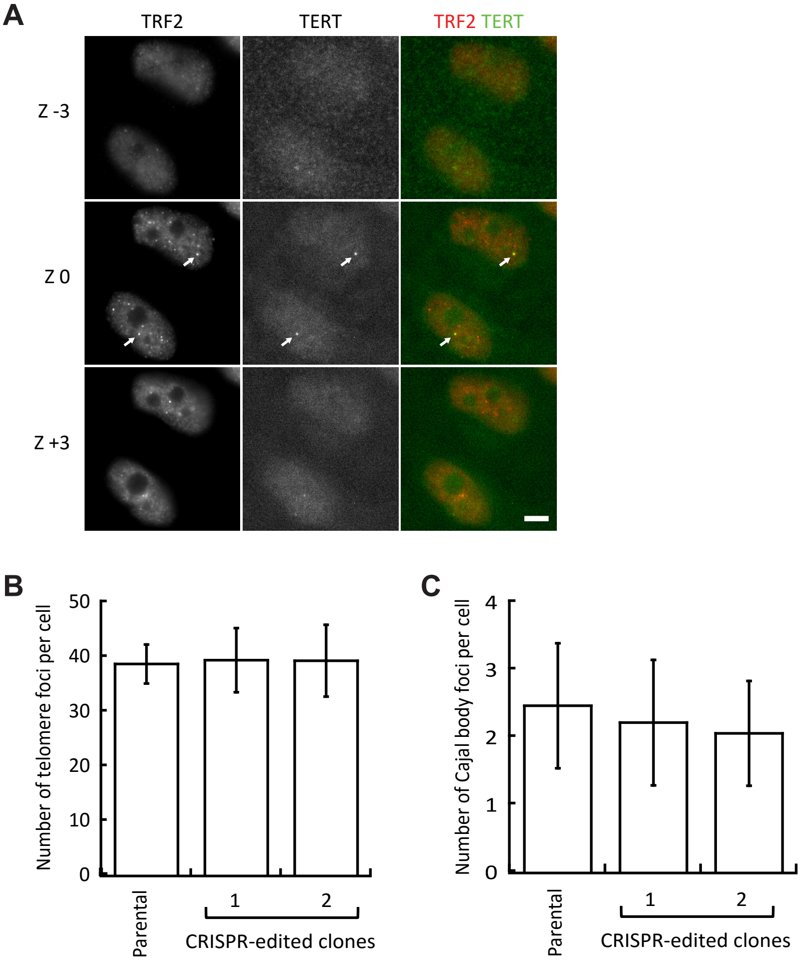
A
TRF2
TERT
TRF2 TERT
Z -3
Z 0
Z +3
B
Number of telomere foci per cell
0
10
20
30
40
50
Parental
1
2
CRISPR-edited clones
C
Number of Cajal body foci per cell
0
1
2
3
4
Parental
1
2
CRISPR-edited clones

Figure S4

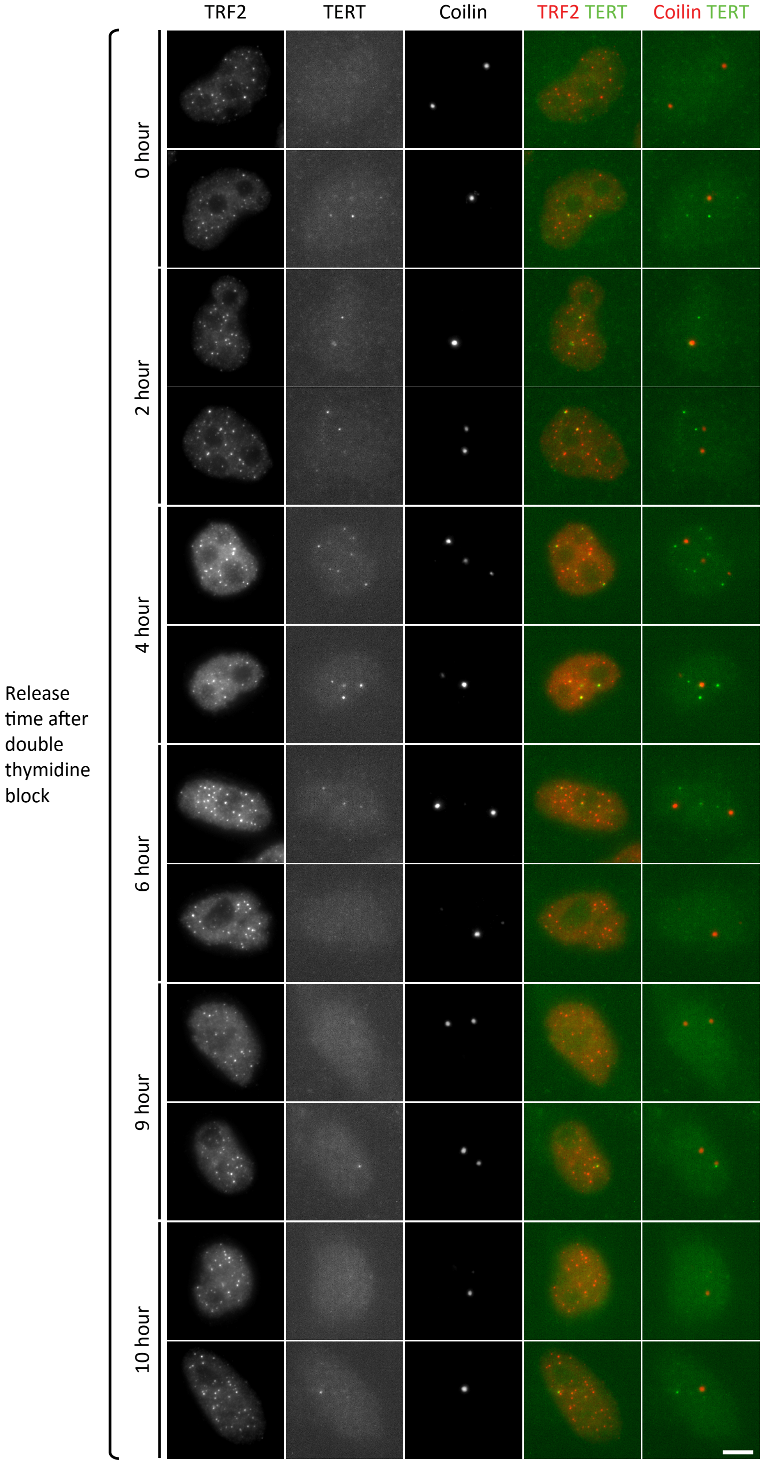

TRF2
TERT
Coilin
TRF2 TERT
Coilin TERT
Release time after double thymidine block
0 hour
2 hour
4 hour
6 hour
9 hour
10 hour

Figure S5

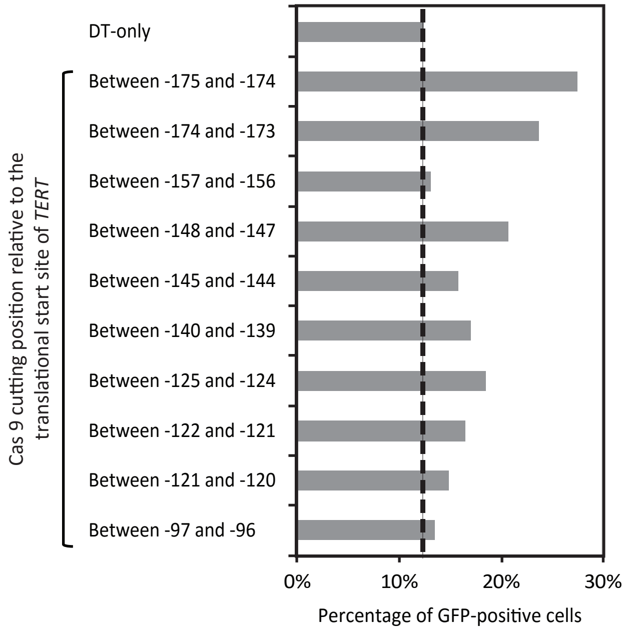
DT-only
Between -175 and -174
Between -174 and -173
Between -157 and -156
Between -148 and -147
Between -145 and -144
Between -140 and -139
Between -125 and -124
Between -122 and -121
Between -121 and -120
Between -97 and -96
Cas 9 cutting position relative to the translational start site of TERT
0%
10%
20%
30%
Percentage of GFP-positive cells

Figure S6

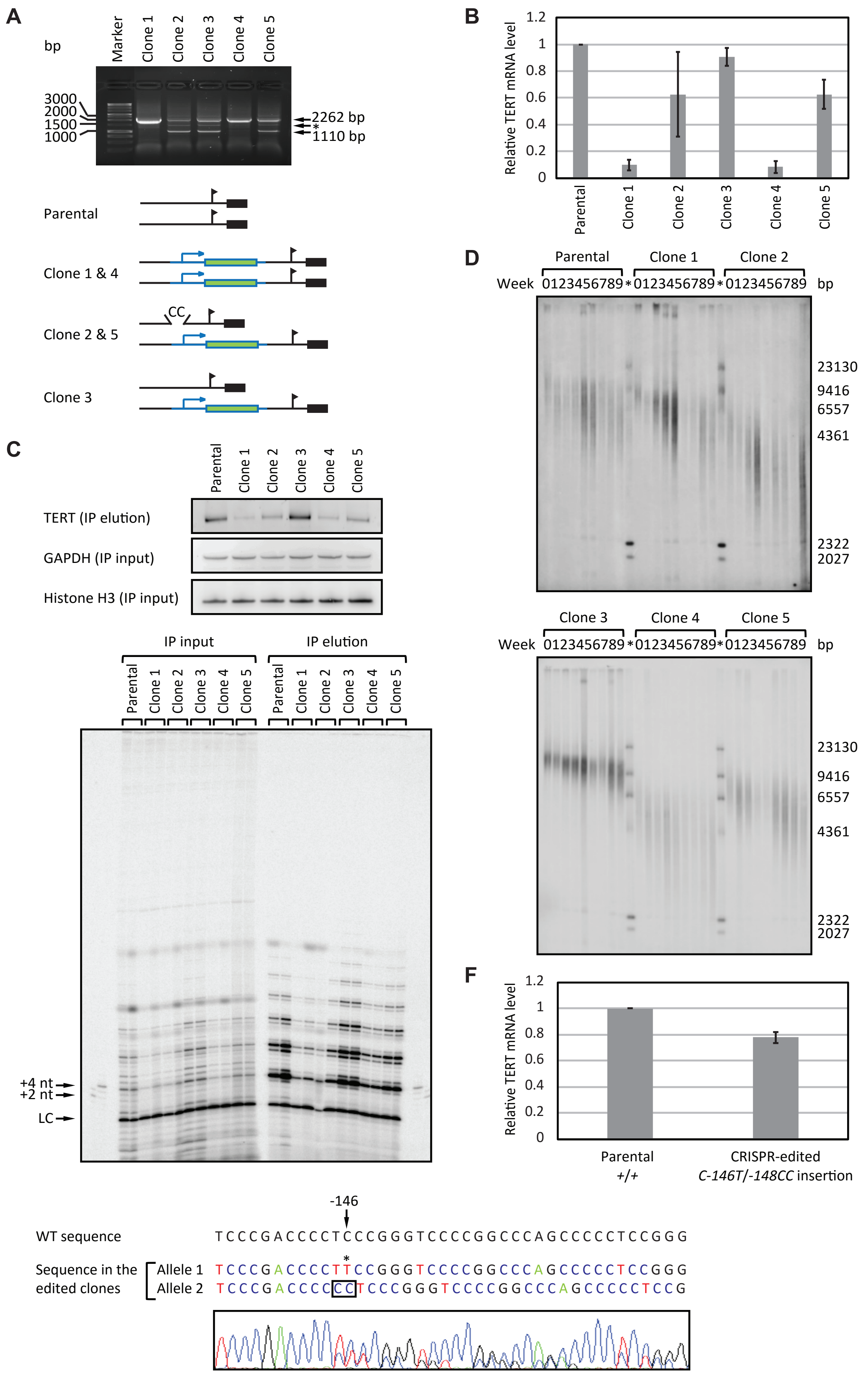
A
bp
Marker
Clone 1
Clone 2
Clone 3
Clone 4
Clone 5
3000
2000
1500
1000
2262 bp
*
1110 bp
Parental
Clone 1 & 4
CC
Clone 2 & 5
Clone 3
B
Relative TERT mRNA level
1.2
1
0.8
0.6
0.4
0.2
0
Parental
Clone 1
Clone 2
Clone 3
Clone 4
Clone 5
C
TERT (IP elution)
GAPDH (IP input)
Histone H3 (IP input)
IP input
IP elution
+4 nt
+2 nt
LC
D
Parental
Clone 1
Clone 2
Week 0123456789*0123456789*0123456789 bp
23130
9416
6557
4361
2322
2027
Clone 3
Clone 4
Clone 5
Week 0123456789*0123456789*0123456789 bp
23130
9416
6557
4361
2322
2027
F
Relative TERT mRNA level
1.2
1
0.8
0.6
0.4
0.2
0
Parental
+/+
CRISPR-edited
C-146T/-148CC insertion
E
-146
WT sequence
TCCCGACCCCTCCCGGGTCCCCGGCCCAGCCCCCTCCGGG
Sequence in the edited clones
Allele 1
Allele 2
*
TCCCGACCCCTTCCGGGTCCCCGGCCCAGCCCCCTCCGGG
TCCCGACCCCCCTCCCGGGTCCCCGGCCCAGCCCCCTCCG
