# Supplementary material for: A novel two-step genome editing strategy with CRISPR-Cas9 provides new insights into telomerase action and TERT gene expression
Source: Genome Biol. 2015 Nov 10;16:231. doi: 10.1186/s13059-015-0791-1 (PMC4640169; doi:10.1186/s13059-015-0791-1)
Supplement: Additional file 2: — A figure showing the TERT copy number analysis for various cell lines generated in this study. (PDF 226 kb) [file 13059_2015_791_MOESM2_ESM.pdf]

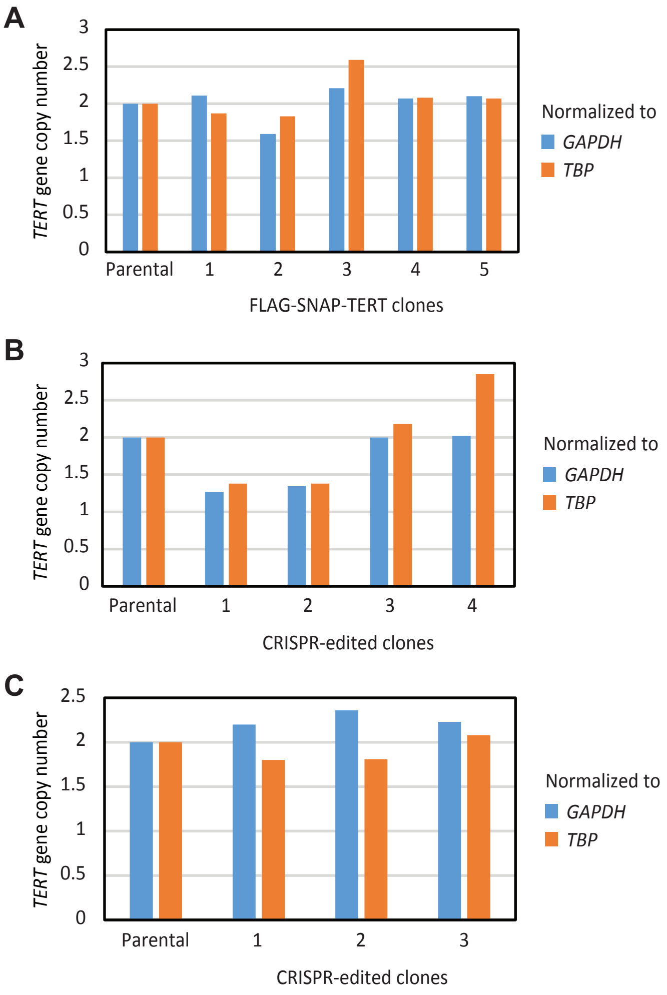

Copy number variation analysis of the *TERT* gene in the CRISPR-edited clones.

Copy number of the *TERT* gene in (A) HEK 293 clones with FLAG-SNAP-TERT, (B) HEK 293T clones with modified *TERT* promoter, and (C) SCaBER clones with modified *TERT* promoter were performed as described in Materials and Methods with the *GAPDH* gene or the *TBP* gene as internal control. In (B), Clone 1 and 2 have lost a copy of the *TERT* gene. Clone 3 and 4 correspond to the clones analysed in Fig S6F.
