# Supplementary material for: A novel two-step genome editing strategy with CRISPR-Cas9 provides new insights into telomerase action and TERT gene expression
Source: Genome Biol. 2015 Nov 10;16:231. doi: 10.1186/s13059-015-0791-1 (PMC4640169; doi:10.1186/s13059-015-0791-1)
Supplement: Additional file 3: — Data file 3, is a detailed description of data files 4-15. Data files 4-15, which are maximum intensity projections of images for all cell biological experiments, including images of an experiment using a FLAG antibody to detect FLAG-SNAP-TERT in HeLa cells. (ZIP 43134 kb) [file 13059_2015_791_MOESM3_ESM.zip › 13059_2015_791_Additional file 3 data file 3.docx]

**Additional Files 7-9.** Subcellular localization of FLAG-SNAP-TERT, related to figure 3.

IF analysis of fixed HeLa cells expressing FLAG-SNAP-TERT. The SNAP-tag was labelled with SNAP-Cell^®^ 647-SiR dye. Telomeres and Cajal bodies were stained with antibodies against TRF2 (523) and coilin (435), respectively. Edited cells but not parental cells showed FLAG-SNAP-TERT foci that co-localized with telomeres and Cajal bodies. Two independent clones expressing FLAG-SNAP-TERT were used to generate the images shown. (100x magnification, 0.064 µm/pixel)

**Additional Files 10-11.** Subcellular localization of FLAG-SNAP-TERT during G1- and S-phase, related to figure 4A.

IF analysis of fixed HeLa cells expressing FLAG-SNAP-TERT. The SNAP-tag was labelled with SNAP-Cell^®^ 647-SiR dye. Telomeres and Cajal bodies were stained with antibodies against TRF2 (523) and coilin (435), respectively. Telomerase is detectable at telomeres in S-phase cells but not in G1-cells. (60x magnification, 0.108 µm/pixel)

**Additional Files 12-17.** Subcellular localization of FLAG-SNAP-TERT throughout S-phase, related to figure 4E.

IF analysis of fixed HeLa cells expressing FLAG-SNAP-TERT. The SNAP-tag was labelled with SNAP-Cell^®^ 647-SiR dye. Telomeres and Cajal bodies were stained with antibodies against TRF2 (523) and coilin (435), respectively. The frequency of TERT co-localization with telomeres increases throughout S-phase, peaking at ~4hours into S-phase. Images are derived from repeat 1 of the experiment. (100x magnification, 0.064 µm/pixel)

**Additional Files 18.** Subcellular localization of FLAG-SNAP-TERT.

IF analysis of fixed HeLa cells expressing FLAG-SNAP-TERT. FLAG-SNAP-TERT was detected using an FLAG-antibody (FITC) and telomeres were stained using a TRF2-antibody (CY5). The FLAG-antibody produces background foci in cell nuclei making reliable co-localization with telomeres hard to detect. (100x magnification, 0.064 µm/pixel)
