# Supplementary figures and images for: A novel two-step genome editing strategy with CRISPR-Cas9 provides new insights into telomerase action and TERT gene expression
Source: Genome Biol. 2015 Nov 10;16:231. doi: 10.1186/s13059-015-0791-1 (PMC4640169; doi:10.1186/s13059-015-0791-1)

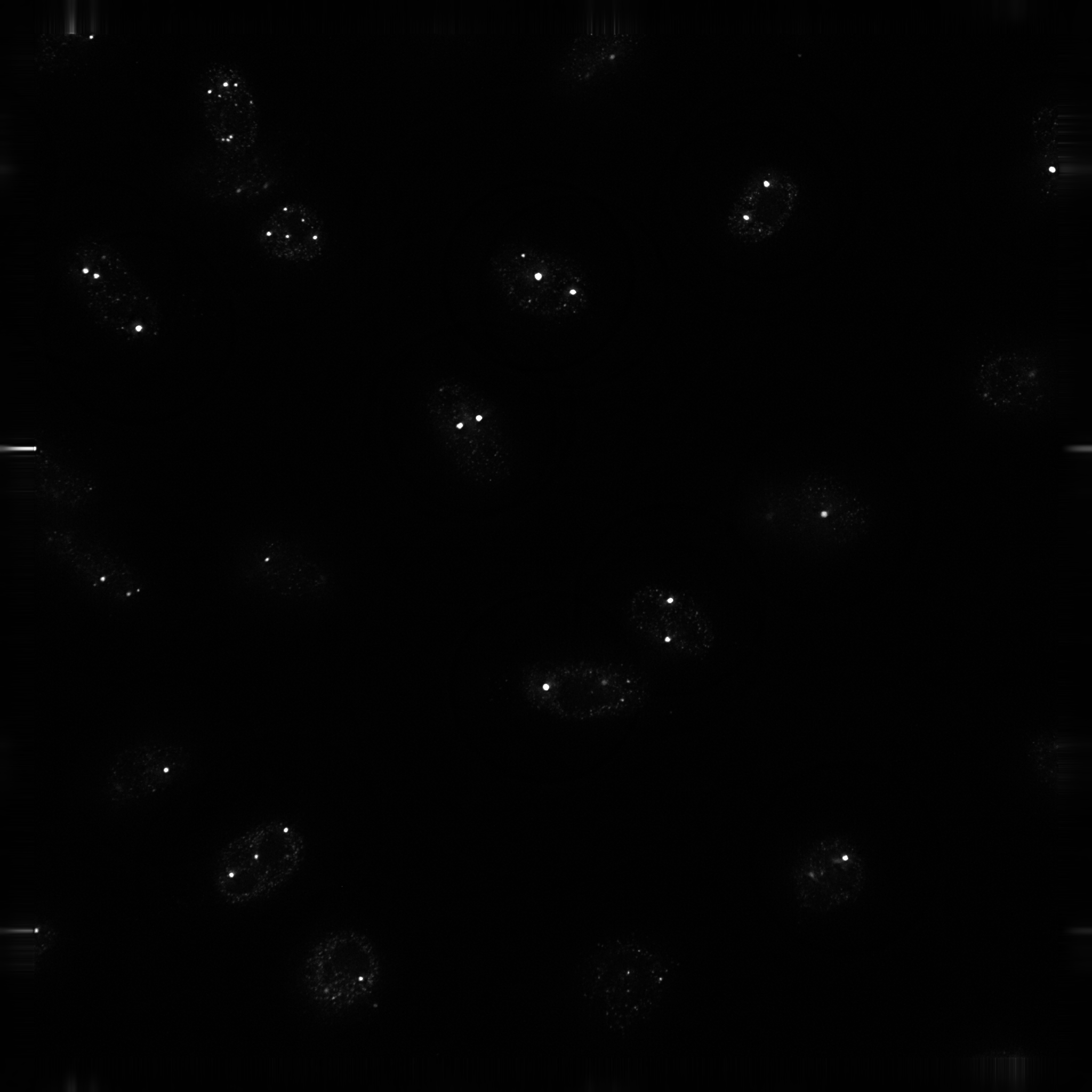

Supplement: Additional file 3: — Data file 3, is a detailed description of data files 4-15. Data files 4-15, which are maximum intensity projections of images for all cell biological experiments, including images of an experiment using a FLAG antibody to detect FLAG-SNAP-TERT in HeLa cells. (ZIP 43134 kb) [file 13059_2015_791_MOESM3_ESM.zip › Fig3_Clone1 - 4.tif]

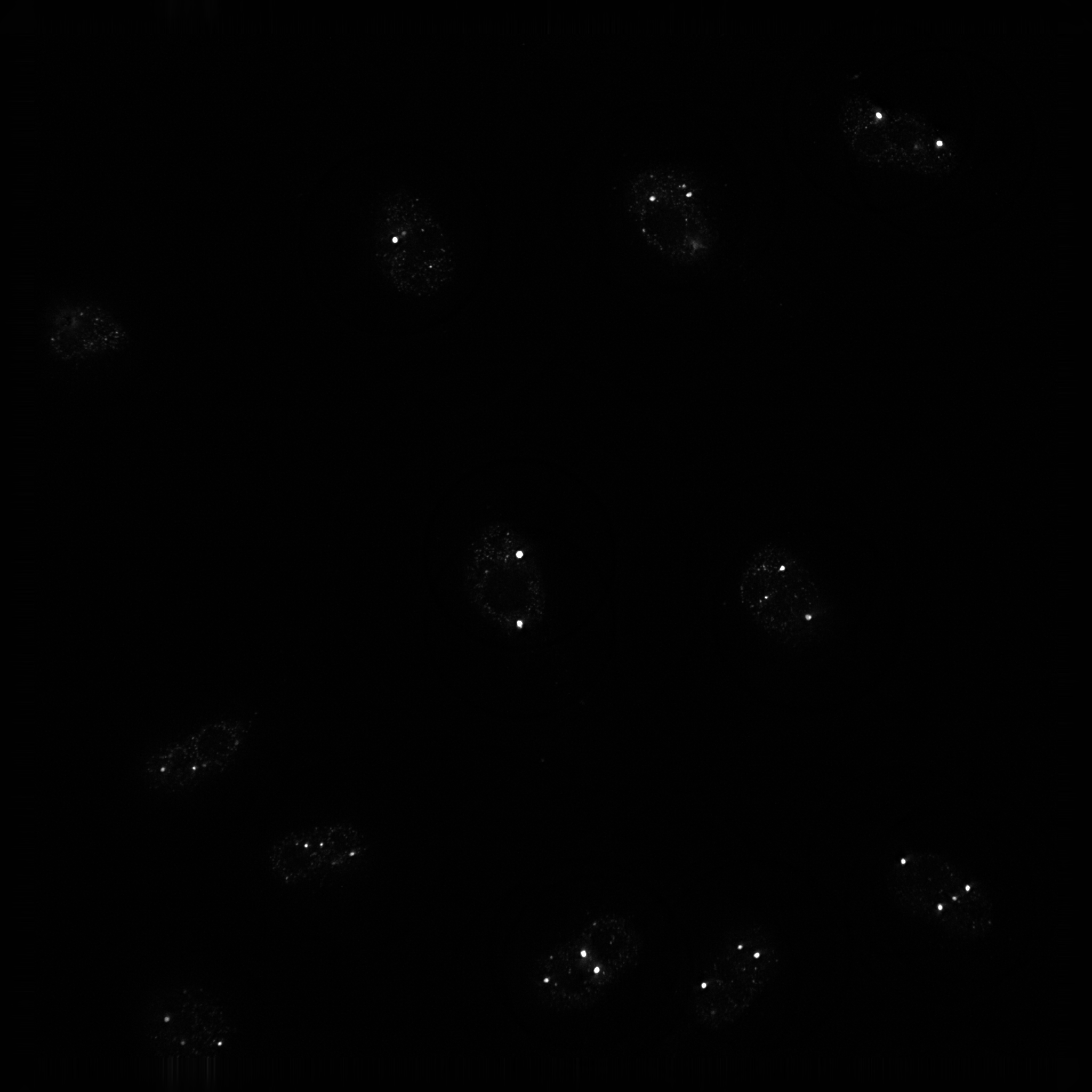

Supplement: Additional file 3: — Data file 3, is a detailed description of data files 4-15. Data files 4-15, which are maximum intensity projections of images for all cell biological experiments, including images of an experiment using a FLAG antibody to detect FLAG-SNAP-TERT in HeLa cells. (ZIP 43134 kb) [file 13059_2015_791_MOESM3_ESM.zip › Fig3_Clone2 - 5.tif]

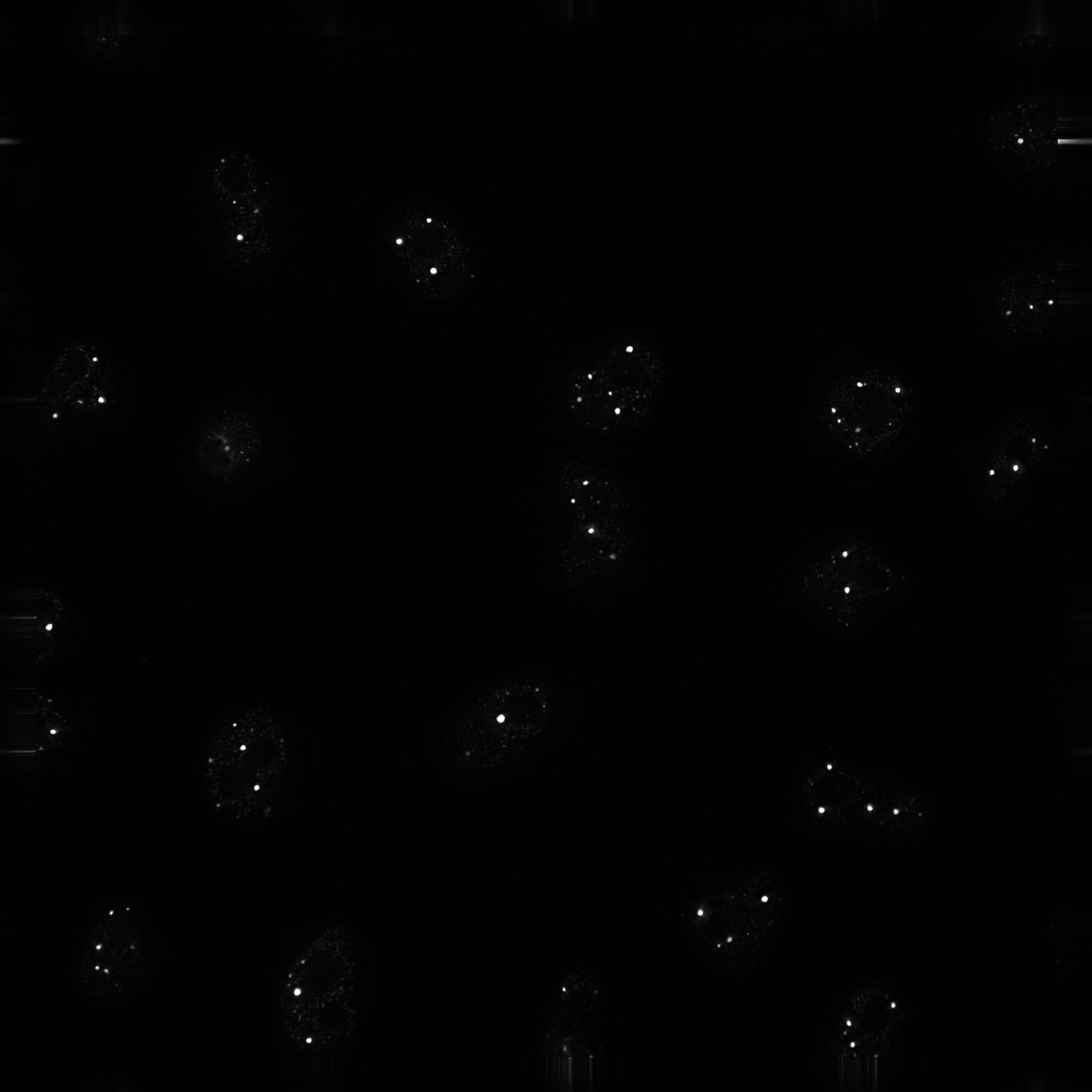

Supplement: Additional file 3: — Data file 3, is a detailed description of data files 4-15. Data files 4-15, which are maximum intensity projections of images for all cell biological experiments, including images of an experiment using a FLAG antibody to detect FLAG-SNAP-TERT in HeLa cells. (ZIP 43134 kb) [file 13059_2015_791_MOESM3_ESM.zip › Fig3_HeLa - 3.tif]

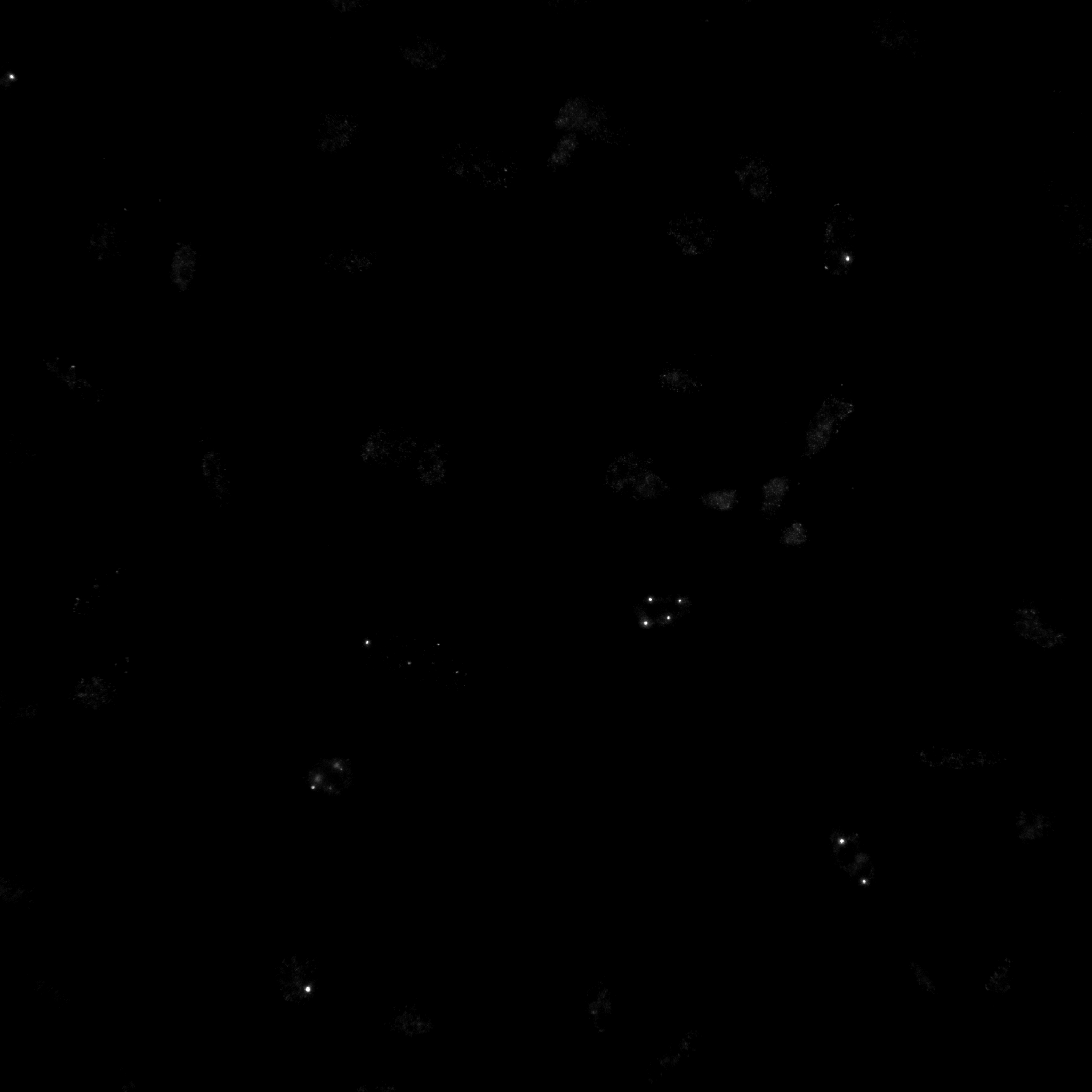

Supplement: Additional file 3: — Data file 3, is a detailed description of data files 4-15. Data files 4-15, which are maximum intensity projections of images for all cell biological experiments, including images of an experiment using a FLAG antibody to detect FLAG-SNAP-TERT in HeLa cells. (ZIP 43134 kb) [file 13059_2015_791_MOESM3_ESM.zip › Fig4A_G1 - 6.tif]

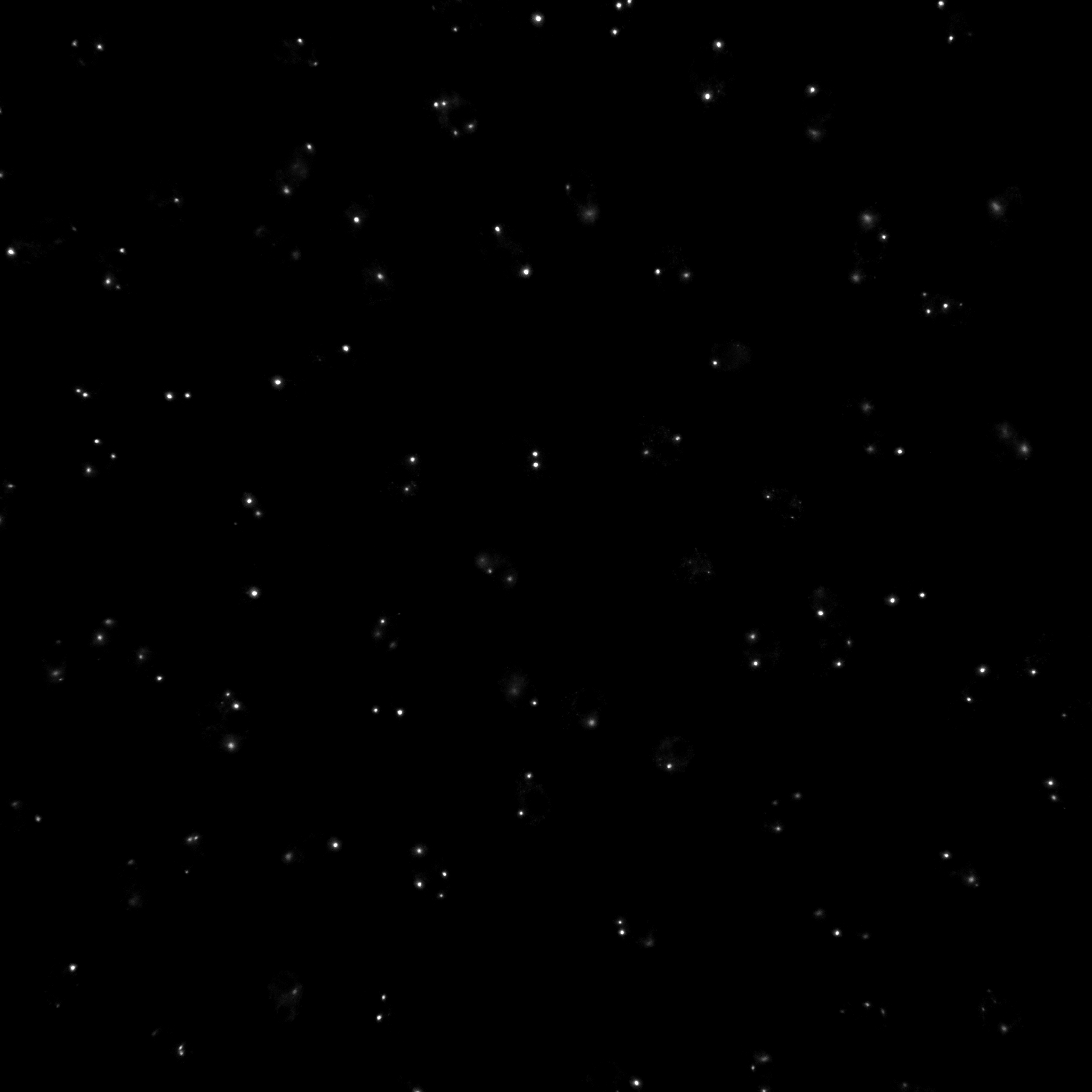

Supplement: Additional file 3: — Data file 3, is a detailed description of data files 4-15. Data files 4-15, which are maximum intensity projections of images for all cell biological experiments, including images of an experiment using a FLAG antibody to detect FLAG-SNAP-TERT in HeLa cells. (ZIP 43134 kb) [file 13059_2015_791_MOESM3_ESM.zip › Fig4A_S - 7.tif]

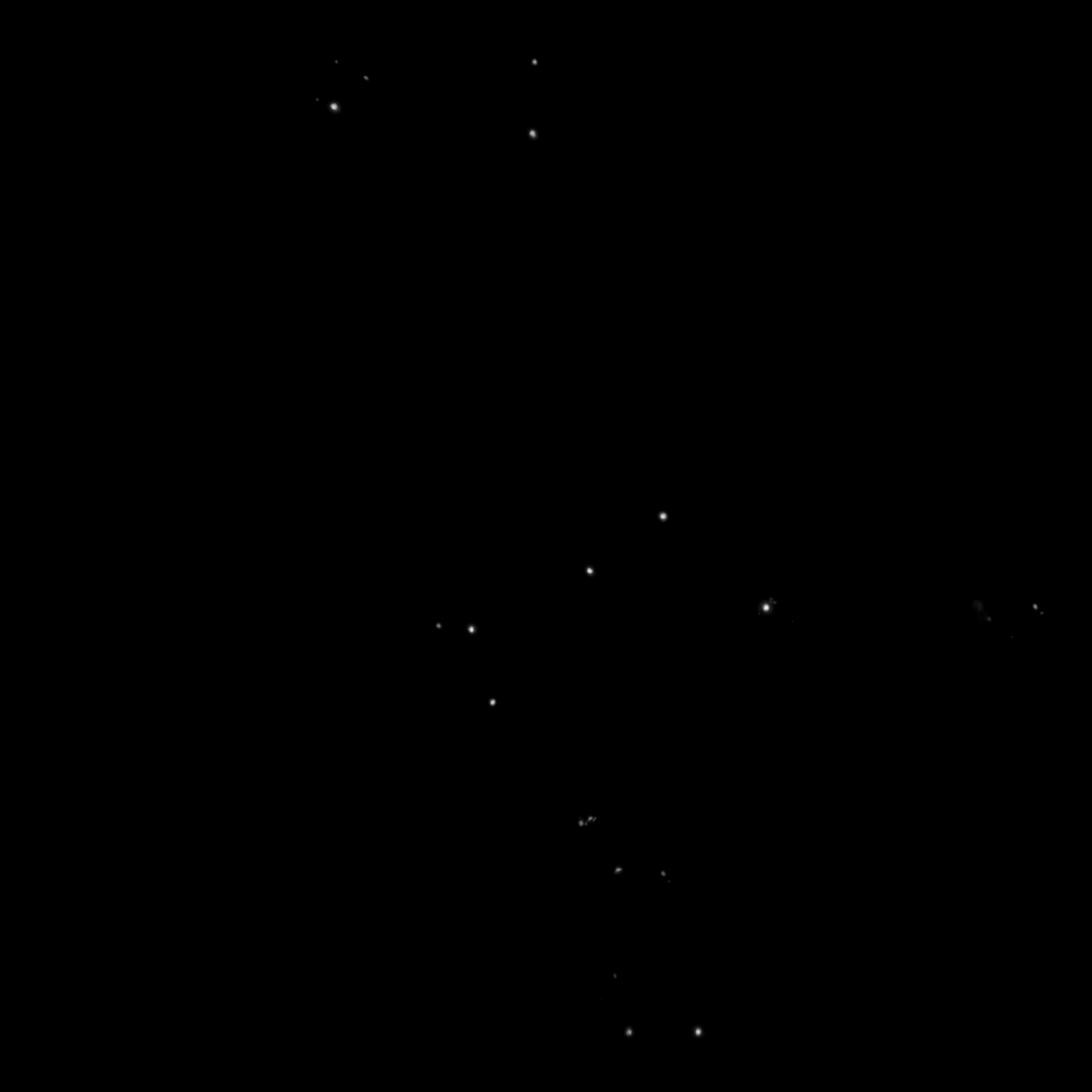

Supplement: Additional file 3: — Data file 3, is a detailed description of data files 4-15. Data files 4-15, which are maximum intensity projections of images for all cell biological experiments, including images of an experiment using a FLAG antibody to detect FLAG-SNAP-TERT in HeLa cells. (ZIP 43134 kb) [file 13059_2015_791_MOESM3_ESM.zip › Fig4E_0h - 8.tif]

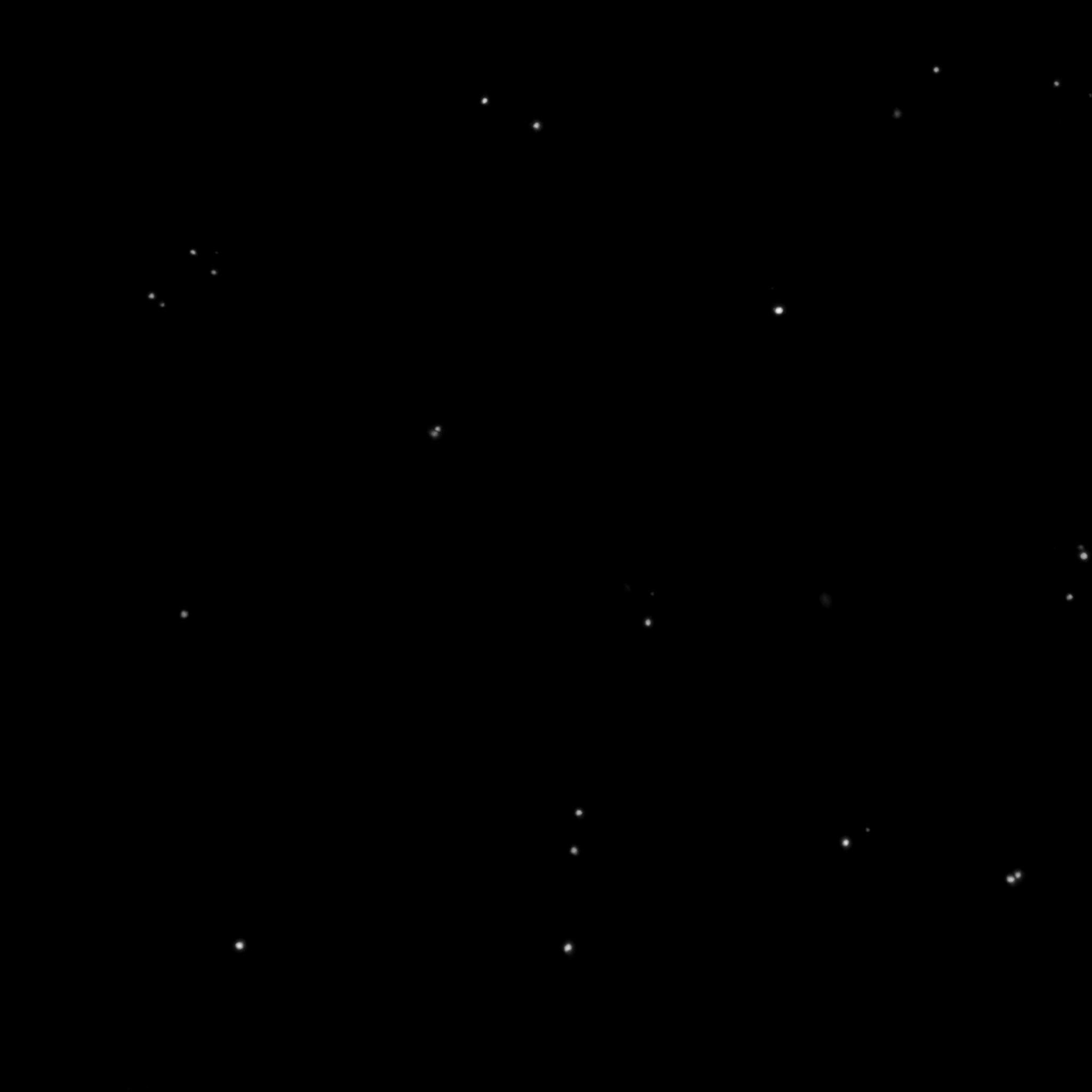

Supplement: Additional file 3: — Data file 3, is a detailed description of data files 4-15. Data files 4-15, which are maximum intensity projections of images for all cell biological experiments, including images of an experiment using a FLAG antibody to detect FLAG-SNAP-TERT in HeLa cells. (ZIP 43134 kb) [file 13059_2015_791_MOESM3_ESM.zip › Fig4E_10h - 13.tif]

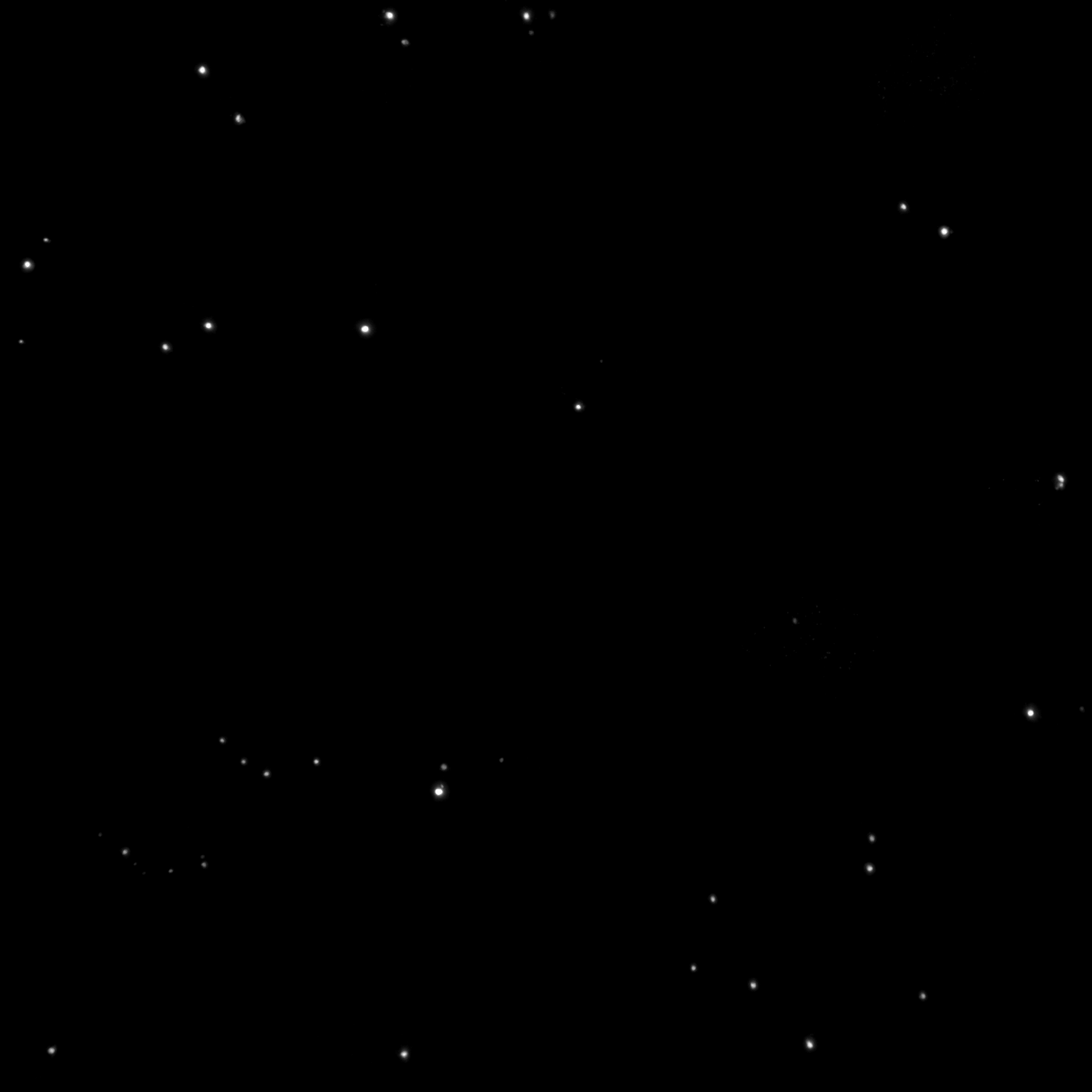

Supplement: Additional file 3: — Data file 3, is a detailed description of data files 4-15. Data files 4-15, which are maximum intensity projections of images for all cell biological experiments, including images of an experiment using a FLAG antibody to detect FLAG-SNAP-TERT in HeLa cells. (ZIP 43134 kb) [file 13059_2015_791_MOESM3_ESM.zip › Fig4E_2h - 9.tif]

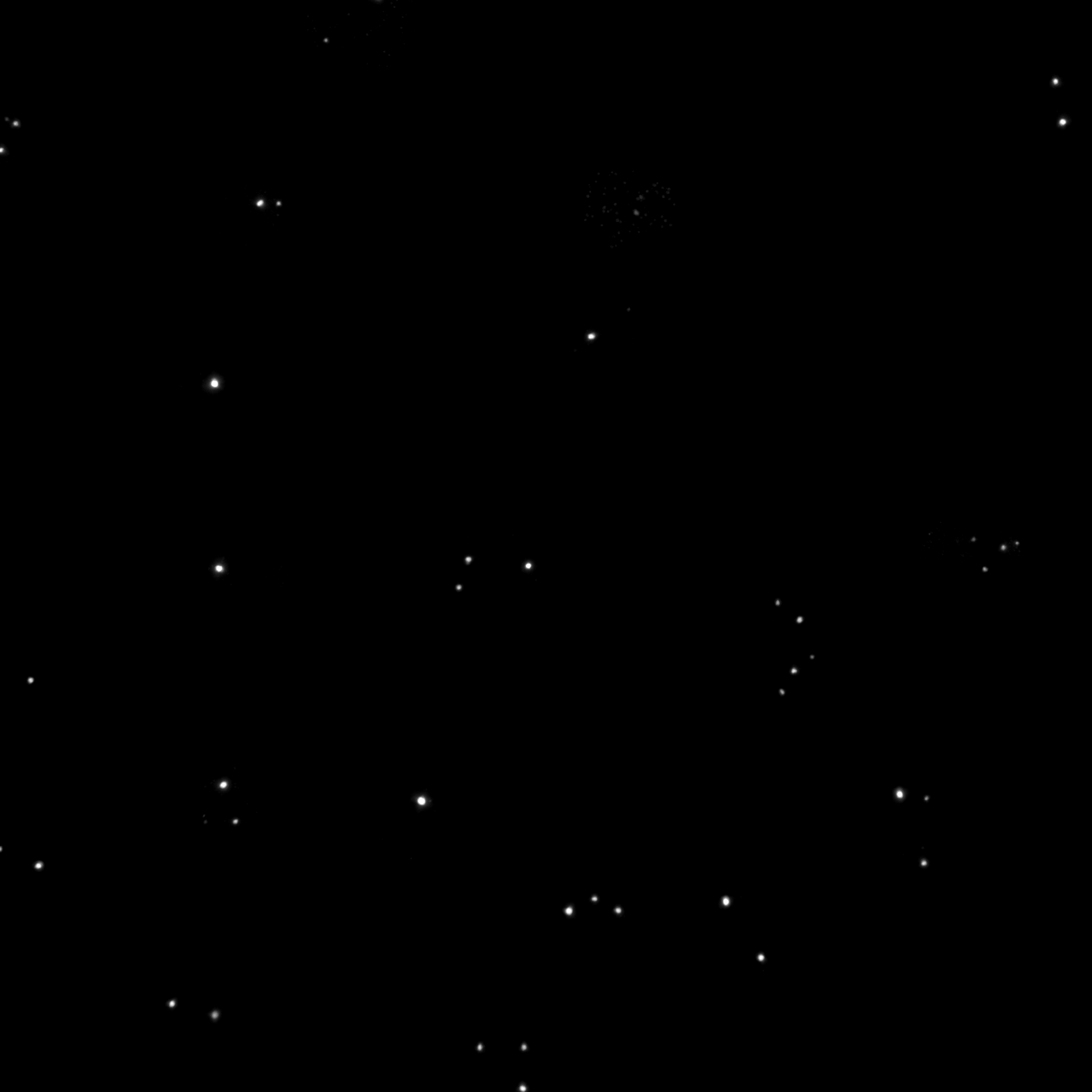

Supplement: Additional file 3: — Data file 3, is a detailed description of data files 4-15. Data files 4-15, which are maximum intensity projections of images for all cell biological experiments, including images of an experiment using a FLAG antibody to detect FLAG-SNAP-TERT in HeLa cells. (ZIP 43134 kb) [file 13059_2015_791_MOESM3_ESM.zip › Fig4E_4h - 10.tif]

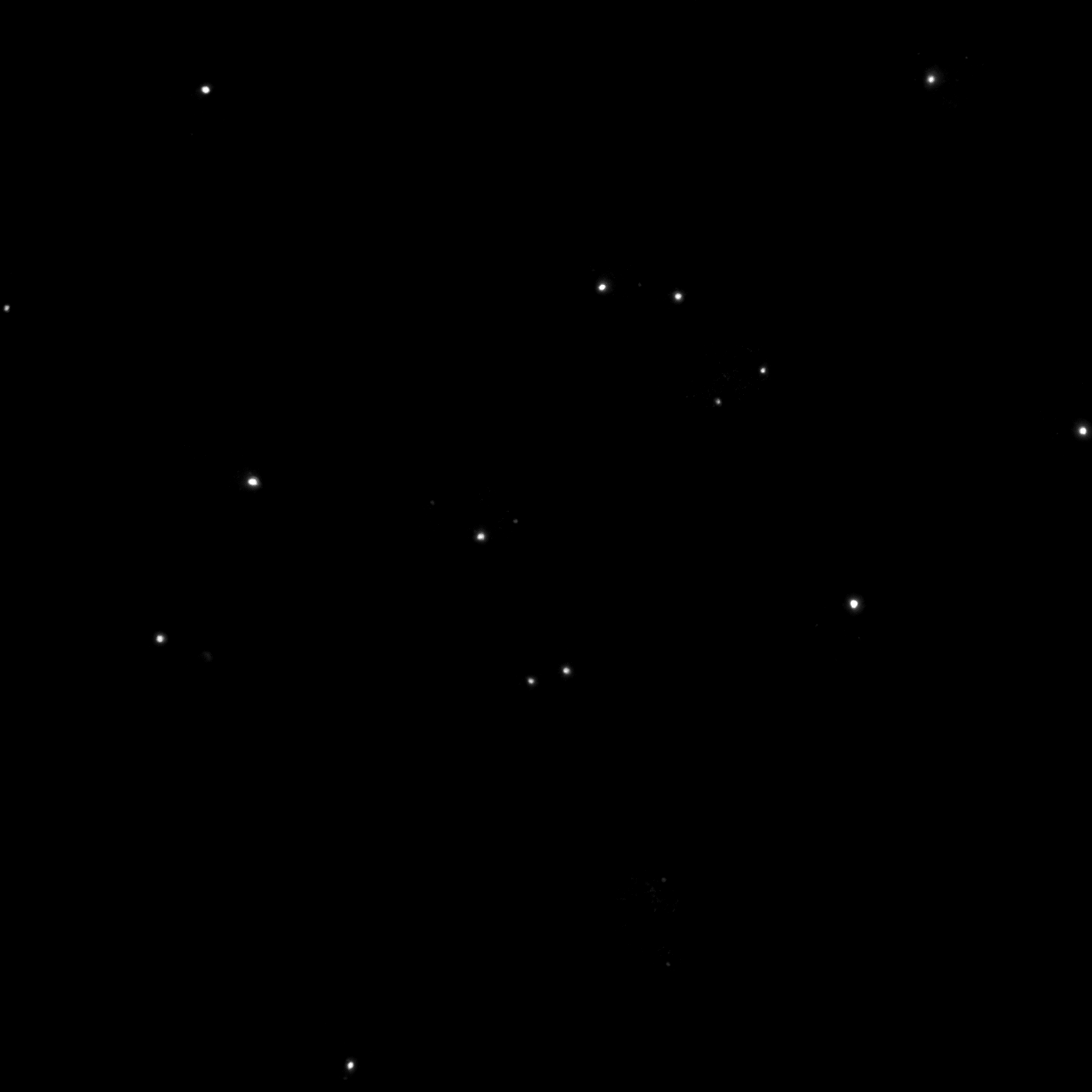

Supplement: Additional file 3: — Data file 3, is a detailed description of data files 4-15. Data files 4-15, which are maximum intensity projections of images for all cell biological experiments, including images of an experiment using a FLAG antibody to detect FLAG-SNAP-TERT in HeLa cells. (ZIP 43134 kb) [file 13059_2015_791_MOESM3_ESM.zip › Fig4E_6h - 11.tif]

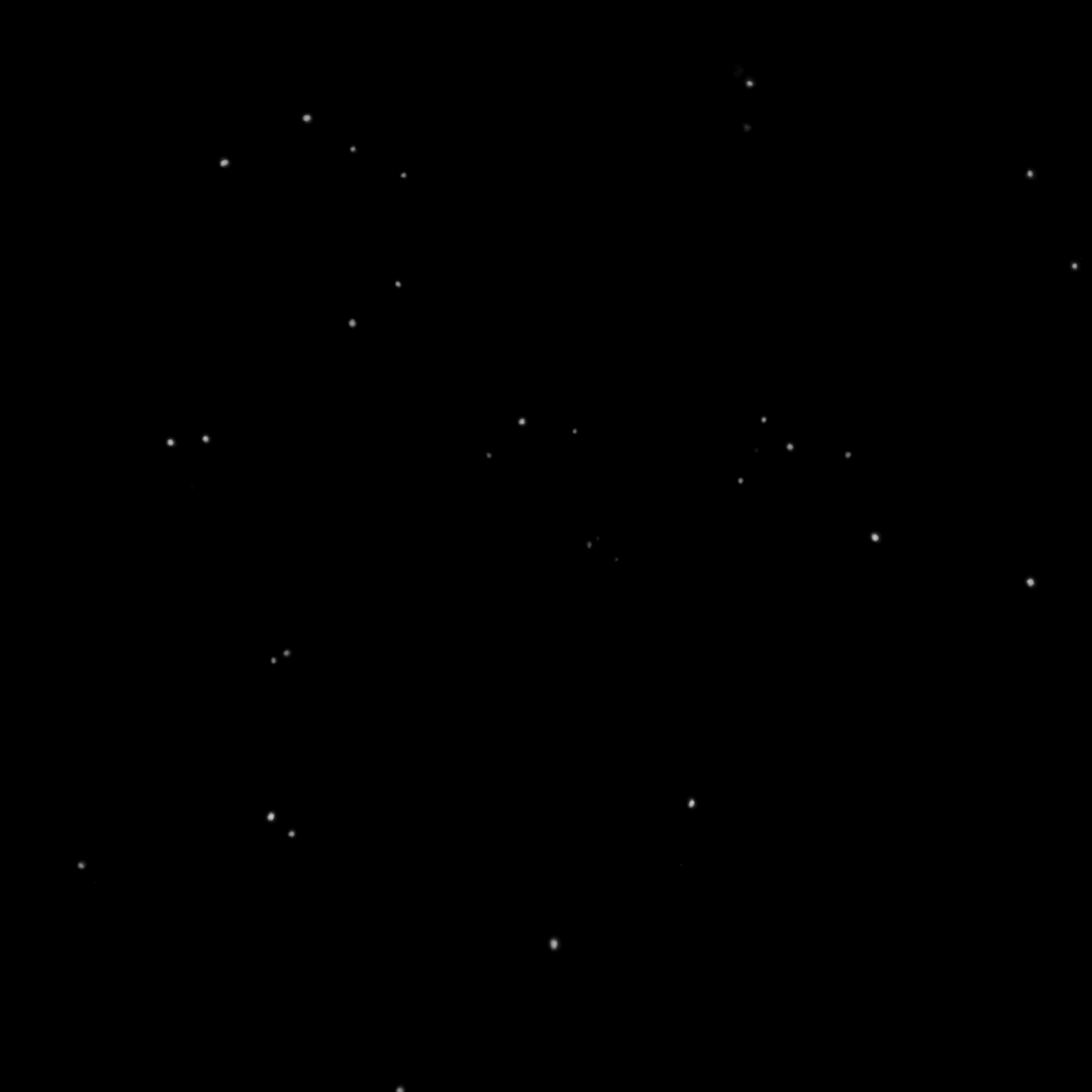

Supplement: Additional file 3: — Data file 3, is a detailed description of data files 4-15. Data files 4-15, which are maximum intensity projections of images for all cell biological experiments, including images of an experiment using a FLAG antibody to detect FLAG-SNAP-TERT in HeLa cells. (ZIP 43134 kb) [file 13059_2015_791_MOESM3_ESM.zip › Fig4E_9h - 12.tif]

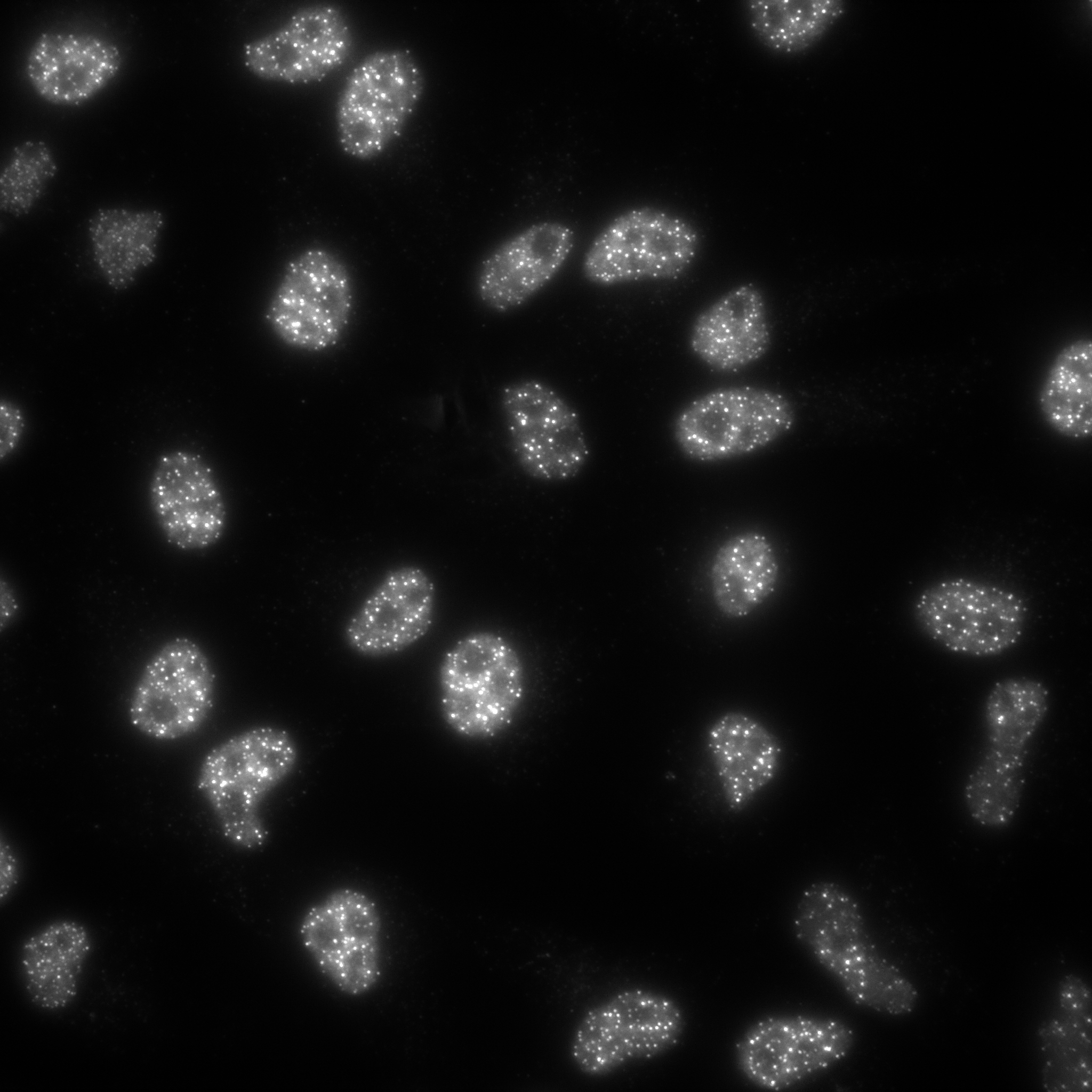

Supplement: Additional file 3: — Data file 3, is a detailed description of data files 4-15. Data files 4-15, which are maximum intensity projections of images for all cell biological experiments, including images of an experiment using a FLAG antibody to detect FLAG-SNAP-TERT in HeLa cells. (ZIP 43134 kb) [file 13059_2015_791_MOESM3_ESM.zip › FLAG_detection - 14.tif]
